# Interventions on Barriers to the Participation of Adolescents in Physical Activity: A Systematic Review

**DOI:** 10.3390/ijerph22060881

**Published:** 2025-05-31

**Authors:** Lauryane Fonseca Terra, Leonardo Mateus Teixeira de Rezende, Regina Márcia Ferreira Silva, Woska Pires da Costa, Vicente Miñana-Signes, Matias Noll, Priscilla Rayanne E. Silva

**Affiliations:** 1 Department of Nutrition, Universidade Federal de Viçosa, Rio Paranaíba 38810-000, MG, Brazil; 2 Public Health Department, Instituto Federal Goiano, Ceres 76300-000, GO, Brazil; leomtr.efi@gmail.com (L.M.T.d.R.); regina.silva@ifg.edu.br (R.M.F.S.); matias.noll@ifgoiano.edu.br (M.N.); 3 Instituto Federal de Goiás, Itumbiara 75525-000, GO, Brazil; 4 Research Department, Instituto Federal Goiano, Morrinhos 75650-000, GO, Brazil; woska.costa@ifgoiano.edu.br; 5 Academic Unit of Physical Education, Body Languages Didactics Department, Universitat de València, 46010 Valencia, Spain; v.minanasignes@edu.gva.es; 6 Universidade Federal de Goiás, Goiânia 74690-900, GO, Brazil; 7 Departamento de Obstetrícia e Medicina, Universidade de São Paulo, São Paulo 05508-070, SP, Brazil

**Keywords:** primary prevention, obstacles, participation in physical activity, physical inactivity, adolescence

## Abstract

This review aimed to assess the effectiveness of interventions used to minimize barriers to participation in physical activity (PA) among adolescents. This systematic literature review followed the Preferred Reporting Items for Systematic Reviews and Meta-Analyses 2020 guidelines. Searches were conducted across five databases: PubMed, SPORTDiscus, Embase, Scopus, and Web of Science. Thirty-four studies evaluated interventions to overcome barriers to participation in PA, with a focus on lack of motivation, encouragement, and support, as well as intrapersonal, interpersonal, and environmental barriers. Most interventions were conducted in developed countries, with an emphasis on girls, reflecting efforts to address health inequities. The interventions, conducted in schools, included workshops, training programs, lectures, goal setting, and practical activities, lasting from four weeks to four years. Twenty-seven studies (79.4%) reported positive impacts on PA participation, particularly in interventions addressing psychosocial, psychological, and behavioral barriers. The most effective interventions combined theory (through educational approaches) and practice (practical PA activities). These findings contribute to understanding barriers leading to physical inactivity and provide insights for developing or replicating initiatives to improve PA levels among adolescents.

## 1. Introduction

Regular participation in physical activity (PA) plays a crucial role in promoting well-being and public health while protecting against the potential adverse consequences of physical inactivity [1,2]. Inactivity is directly linked to non-communicable diseases, such as cancer, diabetes, respiratory, and cardiovascular diseases, representing one of the main behavioral risk factors for the current and future health of adolescents [3,4,5]. In addition, physical inactivity among youth is driven by the Physical Inactivity Triad (PIT), comprising exercise deficit disorder, pediatric dynapenia, and physical illiteracy [6]. To achieve physical, mental, and social health benefits [7,8] individuals must be physically active [9]. The World Health Organization (WHO) recommends that adolescents engage in moderate to vigorous PA for at least 60 min daily [9]. However, despite the importance of this guideline, evidence shows a concerning decline in participation in PA during and after adolescence, with approximately three out of four being considered inactive [10,11,12], which is likely to be due to various barriers to the implementation of interventions at a global level [13,14].

Barriers to participation in PA encompass various factors related to individual, behavioral, and environmental characteristics [13] that hinder or prevent the achievement of specific goals. These include environmental aspects (such as the lack of accessible spaces); socioeconomic and demographic factors (age, income, and education level); sociocultural factors (social norms and values); and psychological, emotional, and cognitive aspects (such as lack of time and motivation) [7,15,16,17]. Thus, these barriers are often shaped by the social determinants of health, which influence health outcomes but are not directly related to medical factors, encompassing socioeconomic and environmental conditions [18]. These determinants affect adolescents’ active participation in PA in various socioeconomic settings. In lower socioeconomic contexts, resources such as access to appropriate spaces, time, and opportunities for PA are more limited, leading to poorer health outcomes [18,19]. Additionally, characteristics of PA (such as intensity and accessibility) and behavioral factors, including previous habits and experiences, also affect adherence [13,14,15].

Physical illiteracy, for example, refers to the lack of confidence, competence, and motivation to perform meaningful physical activities with interest and enthusiasm [6]. Recent studies have identified key barriers among adolescents, including lack of time, lack of motivation, lack of family and peer support, laziness, and lack of access to facilities and specific environments [13,20,21,22]. Beyond identifying these barriers, it is essential to evaluate the impact of intervention programs and actions on PA and health outcomes [23]. This understanding will enable the replication of effective interventions aimed at reducing barriers to PA and inform the development of new programs and initiatives.

Considering the prevalent low participation in PA among adolescents [11,14], establishing effective strategies to reduce physical inactivity is crucial. The effectiveness of interventions, such as projects, programs, and actions, relies on segmented parameters that influence behavioral activities among individuals [24,25,26]. Among all the contexts in which PA can be promoted, educational settings have been identified as the most suitable place to intervene [9]. The successful promotion of PA requires interventions tailored to the characteristics, interests, and settings of adolescents to minimize the barriers [22]. Interventions based on goal modification techniques, monitoring, social support, behavioral changes, and outcomes [27] demonstrate superior results [28]. In recent years, different conceptual frameworks have been developed to provide theoretical support for the promotion of healthy habits in educational settings [29], such as the Active School Creation Model [30], which suggests policies and interventions by public administrations in the areas of health, education, and sports to foster the development of physical activity habits. Furthermore, a recent systematic review emphasized a requirement for interventions targeting behavioral change and/or addressing barriers to PA, prioritizing their dimensions [13]. In targeted interventions, digital technologies are increasingly common, both in behavior change techniques and computer-adapted methods, as well as in providing educational information, self-monitoring, and regular motivational feedback [31,32,33]. Therefore, synthesizing findings from studies addressing barrier-focused interventions, despite their scarcity, may prove essential for developing and implementing future effective interventions.

Previous meta-analyses indicate that interventions aimed at increasing PA levels in adolescents generally have small or minimal effects on moderate-intensity PA practice [34,35,36,37,38]. This scenario persists despite the advancement of recommendations on implementation strategies in school and community settings, including the incorporation of PA into school curricula, a measure widely supported in the literature [39]. These findings reinforce the need for critical reviews of the effectiveness of the adopted approaches and their adaptation to different contexts.

Although recent reviews suggest that multicomponent interventions provide greater health and well-being benefits compared to single-component approaches [40,41], there are no results extending beyond movement behaviors, such as cognitive, academic, physical health, and/or psychological outcomes [37]. Furthermore, a recent meta-analysis highlighted modest positive effects of the interventions but also pointed out a high risk of bias in most of the studies analyzed, emphasizing the importance of developing detailed reports on the implementation of actions and their impacts to enhance knowledge and facilitate the replication of effective approaches [37]. The main conclusion of the reviews is that combined interventions show potential, although the evidence base is still insufficient for definitive claims [38]. Thus, considering the sharp decline in PA during adolescence [11], it becomes essential to develop interventions aimed at minimizing the previously identified barriers, as these represent one of the main factors contributing to the alarming reduction in PA among this age group.

Distinct previous systematic reviews, which analyzed interventions to promote PA in adolescents without a specific focus on barriers and in other contexts [37,38,39], this review specifically examines the effectiveness of interventions targeting known barriers to PA. Given the scarcity of studies exploring interventions addressing identified barriers to participation in PA, understanding how barrier-focused interventions influence PA participation is crucial. To our knowledge, this is the first systematic review assessing the effectiveness of interventions aimed at reducing barriers to participation in PA among adolescents. This study addresses two research questions: “What are the characteristics and outcomes of interventions implemented to minimize barriers to PA”? and “What are the most commonly recurring barriers during the implementation of initiatives to minimize barriers to PA?”. This review is particularly relevant considering that physical inactivity is a public health concern requiring prevention, particularly among adolescents. Beyond the significant potential as a strategy to effectively promote PA, this review aims to provide guidance to reduce health disparities and enhance overall public health [42]. Finally, the findings aim to advance the scientific knowledge of effective interventions while supporting institutional managers and the academic community in developing or replicating projects, programs, and initiatives to promote PA among adolescents.

## 2. Methods

### 2.1. Protocol and Registration

This systematic review protocol was registered in PROSPERO (registration number: CRD42022382174) and subsequently published [43]. The study identification, selection, and evaluation followed the Preferred Reporting Items for Systematic Reviews and Meta-Analyses (PRISMA) 2020 guidelines [44]. Ethical approval was not required, as only published articles were analyzed.

### 2.2. Identification and Selection of Studies

Primary studies, including qualitative, quantitative, and/or mixed methods, published in English were selected, with no restrictions on publication date. Searches were conducted in five databases: MEDLINE/PubMed^®^ via the National Library of Medicine^®^ interface, SPORTDiscus^®^ via the EBSCOhost^™^ interface, Embase^™^, Scopus^™^, and Web of Science Core Collection^™^ between 1 February 2023, and 1 August 2024. The keywords for this systematic review were identified through Medical Subject Headings (MeSH terms): “intervention”, “barrier”, “physical activity”, and “adolescent”. MeSH terms and their synonyms (Table S1) were adapted according to the specific requirements of each database (File S1).

The search strategy and eligibility criteria followed the PICO (population, intervention, comparison, outcome) [45,46,47], which is widely used in evidence-based healthcare research. The “P” component included terms representing adolescents aged 10–19 years of both sexes, as defined by the WHO [9]. The “I” component included terms related to interventions aimed at reducing barriers to participation in PA. The “C” component referred to the absence of interventions and the “O” component focused on participation in PA.

For this review, original peer-reviewed studies published in English, with no date restrictions, were eligible for inclusion, provided they implemented interventions aimed at reducing barriers to physical activity in adolescents (aged 10 to 19 years) [9]. The validity of the eligible studies was assessed, and any retraction records were identified using the Scite tool [48].

Interventions conducted in clinical settings (hospitals and/or nursing homes) and those focusing solely on specific populations (such as rural, Indigenous, refugee, and isolated groups) were excluded. Studies with incomplete data, opinion articles, case reports, commentaries, editorials, dissertations, theses, reviews, and cross-sectional studies were also excluded, as well as those that were inaccessible even after attempts to contact the authors. Similarly, studies involving adolescents with physical or mental disabilities or chronic diseases during sampling were excluded, as well as those that included age groups outside adolescence, except when data were presented separately or could be calculated. Additionally, duplicates published in more than one journal were carefully reviewed to avoid redundancies, and studies that included retractions were also excluded. Finally, studies that discussed barriers to physical activity without the implementation of an associated intervention were excluded, and these could only be identified after a full reading of the articles. All the exclusion criteria were carefully outlined in the protocol [43].

After extracting metadata from the databases through the search strategy, the results were imported into the EndNote^™^ X9 software (Clarivate, Philadelphia, PA, USA) to identify and remove duplicates [49]. The review process followed three steps: (1) screening of titles and abstracts; (2) study selection based on eligibility criteria; and (3) full-text review of potentially eligible studies. Two independent researchers (LFT and LMTR), who were trained to screen articles, performed all the steps using the Rayyan software (Rayyan Systems Inc., Cambridge, MA, USA) [50]. To ensure accuracy, the reviewers conducted a cross-check of eligibility, and any discrepancies in study selection were resolved by a third reviewer (PRES), enhancing the reliability of the selection process. At the end of this process, the articles were included in the systematic review. Inter-rater reliability was calculated at each phase using GraphPad Prism 10.1.0^®^ (GraphPad Software, LLC, San Diego, CA, USA), available at: https://www.graphpad.com/quickcalcs/kappa2/ (accessed on 21 Oktober 2024).

### 2.3. Data Extraction and Analysis

The following data were extracted and summarized from the articles included in the systematic review (File S2): (i) author, year, and place or country of study, (ii) barriers, (iii) intervention used and time, (iv) sample, (v) period in which data collection occurred, (vi) type of study, (vii) instrument used for data collection, (viii) type of analysis performed, and (ix) main results. Two independent reviewers (LFT and LMTR) synthesized the results, with discrepancies resolved by a senior third reviewer (PRES).

Following summarization, a descriptive analysis of the results and content analysis were performed based on the intervention attributes of the PA [51]. Content analysis, a communication analysis technique, was used to categorize data, enabling a structured and systematic understanding of the results from the studies included in this systematic review [51,52]. In cases of missing relevant data, we tried to contact the authors [51].

Initially, a comprehensive review of the material was conducted, followed by the identification of results and coding. Categories emerged through descriptive analysis, enabling the logical and coherent organization of findings. This method provided a robust foundation for data interpretation, ensuring scientific rigor and validity in the construction of categories, and accurately reflecting relevant variables and factors identified in the analyzed studies [51]. Furthermore, the methods and effectiveness of each intervention were compared, allowing the assessment of instruments used to reduce the barriers. To aggregate intervention outcomes (File S2) and present them (Table 1 and Table 2), the studies were analyzed based on their proposals considering the main results and conclusions presented in each study. All the studies were categorized into three levels of intervention effectiveness based on Table 3: “Low” for the studies showing no significant increase or negative outcomes in PA—considered an ineffective result, as indicated in Table 3; “Moderate” for those demonstrating limited or inconsistent positive effects (these studies may have addressed relevant barriers, but the intervention effectiveness was not sufficiently strong)—considered an inconsistent result (showing effects but lacking strong evidence) as indicated in Table 3; and “High” for interventions achieving statistically significant improvements in PA (*p* < 0.05 in most studies) and consistent improvements in the levels of PA of the participants—considered a successful result (Table 3), and the main intervention outcomes are presented in the extraction table (File S2). A meta-analysis was not conducted due to substantial heterogeneity in study designs, outcome measures, and intervention types, which precluded the statistical pooling of the results.

### 2.4. Methodological Quality and Risk of Bias

The strength of evidence was assessed using the Grading of Recommendations, Assessment, Development, and Evaluations (GRADE) questionnaire [53,54] in the GRADEpro GDT online software (McMaster University and Evidence Prime, Inc., Hamilton, ON, Canada). The GRADE criteria evaluate the risk of bias, inconsistency of results, indirectness of evidence, imprecision, and publication bias in healthcare research [55]. Quality of evidence was categorized as follows: (a) high, (b) moderate, (c) low, or (d) very low [55].

To assess the risk of bias in quantitative studies, the translated and adapted version of the 27-item Downs and Black was utilized [56]. However, as some items of the questionnaire were not applicable to observational studies, a modified, condensed version (0–16 points) for longitudinal studies was used [57]. Thus, a group of 16 questions (corresponding to Questions 1–3, 5–7, 9–12, 17, 18, 20, 21, 25, 26) was selected for use. Quality scores were calculated for each study and expressed as a percentage of the maximum possible score for the study design [56]. Studies scoring 70% or higher were classified as having a “low risk of bias”, while scores below 70% were considered to indicate a “high risk of bias” [53,54].

The Critical Appraisal Skills Programme Qualitative Research Checklist (CASP) [58] was used to assess the risk of bias in qualitative studies. The evidence was evaluated through ten criteria [59]: (1) clear objectives; (2) methodology appropriate to the objectives; (3) study design appropriate to the objectives; (4) appropriate recruitment strategy; (5) data collection methods appropriate to the research question; (6) researcher–participant relationship; (7) ethical considerations; (8) rigorous analysis; (9) clear presentation and discussion of results; and (10) research contributions and implications to scientific knowledge. Studies were categorized based on their scores as low (0–3 points), moderate (4–7 points), or high quality (8–10 points) [60].

Two independent reviewers (LFT and LMTR) assessed the strength of evidence and the risk of bias, with discrepancies resolved by a third reviewer (PRES). The reviewers underwent preparatory training [61], and all the methodological procedures adhered to the pre-registered protocol [43]. All the studies were examined for declarations of potential conflicts of interest and ethical approval.

## 3. Results

### 3.1. Inter-Rater Reliability

**Figure 1:**
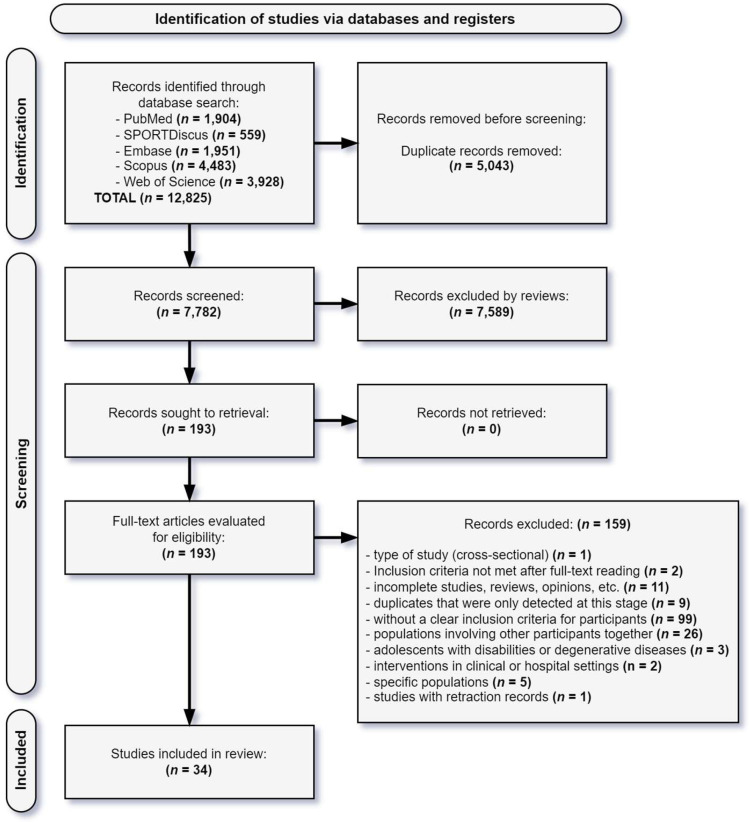
Flow diagram of study identification, screening, and inclusion in the systematic review (PRISMA 2020).

The initial search yielded 12,825 studies. After removing 5043 duplicates and excluding 7589 articles during title and abstract screening, 193 studies remained for full-text review. The process of inclusion of articles in the systematic review is illustrated in the PRISMA 2020 flow diagram (Figure 1). The reviewers demonstrated strong agreement on study eligibility during title and abstract screening (98.3% agreement, κ = 0.74; 95% confidence interval: 0.70–0.78; standard error: 0.022) and near-perfect agreement during full-text review (98.4% agreement, κ = 0.95; 95% confidence interval: 0.89–1.00; standard error: 0.027).

### 3.2. Characteristics of the Selected Studies

During screening, 159 studies that did not meet the predefined inclusion criteria were excluded. After a full-text review, 34 studies that met the eligibility criteria were included (File S2) and synthesized (Table 1). No additional studies were identified through secondary searches of the reference lists in the included studies.

**Table 1:** Main information of the interventions included in this systematic review.

#	Author, Year, and Country of Study	Type of Interventions	Time	Sample	Environments	Methods	Theoretical Framework	Barriers	Use of Electronic Equipment **	Level of Effectiveness
Quantitative										
1 [62]	Aceves-Martins, M. et al. (2022) Spain	Educational approach	12 months	169 (13–16)	School (socioeconomically disadvantaged neighborhoods)	Training and goals	Yes	Lack of encouragement, guidance, and screen time	Yes	Moderate
2 [63]	Andruschko, J. et al. (2018). Australia	Mixed approach	6 months	292 girls (from 7th to 9th grade)	School	Sessions and classes	Yes	Low physical fitness and lack of enjoyment	No	High
3 [64]	Åvitsland, A. et al. (2020). Norway	Mixed approach	29 weeks	1391 (14–15)	School	Classes	Yes	Lack of motivation, self-efficacy, and psychosocial mechanism	No	Moderate
4 [65,66]	Barbosa Filho, V.C. et al. (2016); and *** Barbosa Filho, V.C. et al. (2019). Brazil	Educational approach	4 months	1085 (11–18) students from 6 schools	School (neighborhoods with low HDI 0.170–0.491)	Classes	No	Lack of knowledge and encouragement, screen time, school environment, lack of equipment, and lifestyle	No	High
5 [67]	Bianchi-Hayes, J. et al. (2018). USA	Practical approach	10 weeks	9 (14–16) * overweight or obese	Location (city)	Goals	No	Lack of motivation and parental support	Yes	Low
6 [68]	Chen, Y. et al., (2023). USA	Practical approach	9 weeks	15 (12–17) *	Location (households)	Space and activities	No	Lack of suitable environments and low physical fitness and skills	No	Low
7 [69]	Christiansen, L.B. et al. (2018) Denmark	Educational approach	9 months	2797 (10–13 years old)	School	Workshops, courses, educational materials, planning guides, and lesson plans	No	Lack of self-confidence and lack of motivation and support	Yes	Low
8 [70]	Cook, T.L. et al. (2014). Europe	Educational approach	3 months	536 students (12–17)	International (Austria; Greece; Belgium; Germany; and Sweden).	Training, goals, and activities	Yes	Neighborhood safety, sports facilities in the neighborhood, lack of sports facilities at school, and social support	Yes	High
9 [71]	Dunton, G.F. et al. (2007). California	Mixed approach	9 months	122 girls (14–17)	School	Lectures, activities, and sessions	Yes	Lack of self-efficacy, lack of motivation, time, and enjoyment	No	High
10 [72]	Gråstén, A. et al. (2015). Finland	Educational approach	12 months	847 (12–14)	School	Training, classes, and workshops	No	Lack of motivation and lack of access to environments and equipment	No	Moderate
11 [73]	Jamner, M.S. et al. (2004). California	Mixed approach	4 months	58 sedentary girls	School	Lectures, goals, and classes	No	Psychosocial factors, lack of social support, and lack of enjoyment when participating in PA	No	Moderate
12 [74]	Lennox, A. et al. (2013). South Africa	Practical approach	6 months	252 (aged 14.8)	School (socioeconomically disadvantaged neighborhood)	Sessions	No	Lack of incentive	No	Moderate
13 [75]	Lindgren, E.C. et al. (2011). Sweden	Practical approach	6 months	110 girls (13–19)	School (socioeconomically disadvantaged)	Sessions and activities	No	Lack of self-efficacy	No	High
14 [76]	Sanaeinasab, H. et al. (2012). Iran	Educational approach	2 months	165 (13–15)	School	Lectures, workshops, and competitions	No	Lack of encouragement and support	Yes	High
15 [77]	Taymoori, P. et al. (2008). Iran	Educational approach	6 months	161 girls (9th and 10th grade)	School	Lectures, goals, and classes	No	Lack of support, knowledge, and self-efficacy	No	Moderate
16 [78]	Tennfjord, M.K. et al. (2023). Norway	Educational approach	4 years	1221 (11–12)	School	Classes	No	Psychosocial problems (increased well-being and self-concept)	No	Low
17 [79]	Verswijveren, S.J.J.M. et al. (2022). Australia	Educational approach	3 months	273 (≥13) physically inactive	School (socioeconomically disadvantaged)	Goals and lessons	Yes	Self-efficacy, lack of support, lack of self-regulation strategies, and lack of enjoyment	Yes	Low
18 [80]	Wilson, D.K. et al. (2011). Columbia	Mixed approach	17 weeks	1563 (10–14 years) (55% women)	School	Goals, activities, and lessons	Yes	Lack of behavioral, social, and environmental skills	Yes	High
**Mixed**										
19 [81,82]	Carlin, A. et al. (2018); e *** Carlin, A. et al., (2019). North Ireland	Mixed approach	12 weeks	199 girls [45 (11–14) in complementary study]	School	Walking sessions	Yes	Lack of motivation, lack of time, and lack of opportunities in the school setting	Yes	High
20 [83]	Corder, K. et al. (2020). North Ireland	Practical approach	12 weeks	1874 (13–14)	School	Lessons and goals	Yes	Lack of support, motivation, time pressure, and self-esteem	No	Low
21 [84]	Corepal, R. et al. (2019). North Ireland	Practical approach	22 weeks	224 (12–14)	School	Goals	Yes	Lack of motivation, support, and mental malaise	Yes	Low
22 [85]	Dudley, D.A. et al. (2010). Australia	Practical approach	3 months	38 low-income girls (11th grade) *	School	Training and sessions	Yes	Lack of enjoyment when participating in PA, lack of support, and low self-perception	Yes	Low
23 [86]	Ferreira Silva, R.M. et al., (2023). Brazil	Educational approach	4 weeks	80 (15.9 ± 1.15)	School	Educational materials	Yes	Lack of knowledge, lack of encouragement, and lifestyle	Yes	Low
24 [87]	Koorts, H. et al. (2020). Australia	Mixed approach	12 weeks	142 (mean age 13.7)	Schools (socio-economically disadvantaged neighborhoods)	Goals and activities	Yes	Lack of awareness, motivation, and incentive	Yes	Moderate
25 [88]	Kroshus, E. et al., (2023). USA	Practical approach	12 months	1076 (11–14)	School	Provision of kits	Yes	Lack of suitable environments and lack of company	No	Low
26 [89]	Lubans, D.R. et al. (2014). Australia	Educational approach	5 months	361 (12.7 ± 0.5) inactive boys	School (socioeconomically disadvantaged neighborhoods)	Sessions, goals, and training	Yes	Lack of motivation and long screen time	Yes	Low
27 [90]	Moore, R. et al., (2024). Inglaterra	Educational approach	3 months	9 (11–13) *	School	Conversation agent	Yes	Lack of confidence and motivation	Yes	Low
28 [91]	Sutherland, R. et al. (2020). Australia	Educational approach	24 months	6476; 49 schools from grades 7th to 9th	School (socioeconomically disadvantaged neighborhoods)	Lessons, plans, and practices	Yes	Lack of support	No	High
**Qualitative**										
29 [92]	Bean, C.N. et al. (2014). Canada	Educational approach	12 months	10 girls (11–14)	Local (socio-economically disadvantaged)	Workshops, awareness talks, and series of sessions	Yes	Lack of support, lack of suitable environments and structures, lack of self-control and motivation	No	High
30 [93]	Drehlich, M. et al. (2020). Australia	Educational approach	12 weeks	124 physically inactive (13–14)	School (socio-economically disadvantaged)	Missions and materials	Yes	Lack of motivation and socio-economic conditions	Yes	Low
31 [94]	Lodewyk, K.R. et al. (2023). Canada	Educational approach	3 months	25 students girls (of 483)	School	Training, sessions, and activities	Yes	Lack of motivation	Yes	High
32 [95]	Mitchell, F. et al. (2015). Scotland	Educational approach	4 months	41 girls (11–16)	School	Workshops and classes	Yes	Lack of motivation, autonomy, and interest	No	High
33 [96]	Pierre, S.T. et al., (2024). USA	Mixed approach	3 years	14 (14.8 ± 1.7)	School	Activities and elective courses for coaches	Yes	Lack of support and encouragement	No	High
34 [97]	Wright, P.M. & Burton, S. (2008). USA	Educational approach	12 months	23 African Americans (mean age of 14.8) from an urban high school	Schools (socio-economically disadvantaged neighborhoods)	Lessons and goals	No	Lack of autonomy, stress, lack of motivation, and skills	No	High

Note: PA stands for physical activity, and HDI stands for human development index. * a sample size of <50 participants is insufficient for consistent results [98,99]. ** for sending messages, brochures, or reminders. *** two articles published in different years but complementary to another article by the same lead author, both referring to the same study. Observation: (1) In the “environments” column, it indicates interventions occurred at different levels (household, neighborhood, city and/or state). (2) “Mixed approach” indicates both theoretical and practical methods were used in intervention design and implementation. (3) Intervention effectiveness is classified into three categories: “Low” for the studies showing no significant increase in PA or negative results; “Moderate” for the studies showing some positive impact but with limited/inconsistent results; and “High” for the studies demonstrating significant and consistent increases in participants’ PA, with statistically significant results and robust approach to addressing identified barriers. The main outcomes for each intervention can be found in the extraction table (File S2) and Table 3.

**Figure 2:**
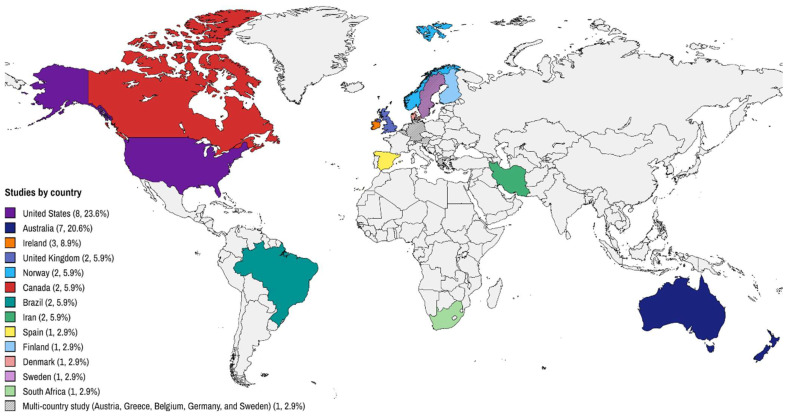
Total number published by geography. Geographic distribution of interven-tion studies worldwide and frequency in percentage (one study covered multiple countries). Created by the authors using Figma® software (Figma, Inc., San Francisco, CA, USA), available at: https://www.figma.com/ (accessed on 21 September 2024).

The studies were published between 2004 and 2024, with 19 (55.9%) published after 2018 [62,63,64,67,68,69,78,79,81,83,84,86,87,88,90,91,93,94,96]. The studies predominantly originated from developed countries, with the United States leading (*n* = 8, 23.6%) [67,68,71,73,80,88,96,97]; followed by Australia (*n* = 7, 20.6%) [63,79,85,87,89,91,93], Ireland (*n* = 3) [81,83,84], Canada (*n* = 2) [92,94], United Kingdom (*n* = 2) [90,95], Norway (*n* = 2) [64,75], and Iran (*n* = 2) [76,77]; and single studies from Spain [62], Denmark [69], Finland [72], and Sweden [75]. In addition to these studies, a comprehensive European study encompassing five countries (Austria, Greece, Belgium, Germany, and Sweden) was identified [70]. Developing nations, including Brazil [65,86] and South Africa [74], were also represented (Table 2). In summary, the geographical distribution of the studies showed that Europe and the Americas each accounted for 35.3% *(n* = 12), followed by Oceania with 20.6% (*n* = 7), while Asia and Africa had lower representation with 5.9% (*n* = 2) and 2.9% (*n* = 1), respectively (Figure 2).

**Table 2:** Characteristics of the studies included in the systematic review.

Characteristics	Categories	Number of Studies (%)
Publication Year	2004–2010	5 (14.7%)
	2011–2017	10 (29.4%)
	2018–2024	19 (55.9%)
Type of Study	Quantitative	18 (52.9%)
	Mixed	10 (29.4%)
	Qualitative	6 (17.7%)
Region		
Oceania	Australian	7 (20.6%)
Europe	Ireland	3 (8.9%)
	Spain	1 (2.9%)
	United Kingdom	2 (5.9%)
	Norway	2 (5.9%)
	Finland	1 (2.9%)
	Denmark	1 (2.9%)
	Sweden	1 (2.9%)
	Austria, Greece, Belgium, Germany, and Sweden *	1 (2.9%)
America	United States	8 (23.6%)
	Canada	2 (5.9%)
	Brazil	2 (5.9%)
Africa	South Africa	1 (2.9%)
Asia	Iran	2 (5.9%)
Sex	Male sex only	1 (2.9%)
	Female sex only	10 (29.4%)
	Both sexes	23 (67.7%)
Sample size	<100	12 (35.3%)
	100–500	12 (35.3%)
	501–1000	2 (5.9%)
	>1000	8 (23.5%)
Sample economic status	Low	13 (38.2%)
	High	–
	Unidentified	21 (61.8%)
Duration of interventions	<1 month	–
	1–6 months	21 (61.7%)
	7–12 months	10 (29.4%)
	>12 months	3 (8.9%)
Implementation in the school environment or through it	Yes No	31 (91.2%) 3 (8.8%)
Electronic Equipment	Yes	16 (47.0%)
	No	18 (53.0%)
Theoretical Frameworks	Yes	20 (58.8%)
	No	14 (41.2%)
Instrument used **	Questionnaire	27 (79.4%)
	Interviews	16 (47.0%)
	Self-reports	14 (41.2%)
	Accelerometry	9 (26.5%)
	Measurement instruments	5 (14.7%)
	Applicative	2 (5.9%)
	Pedometric	2 (5.9%)

Note: * comprehensive study conducted in more than one country; ** more than one instrument used per study.

Sample sizes of the included studies ranged from nine to 2797 adolescents, with variations attributed to methodological approaches. Ten intervention programs (29.4%) exclusively targeted adolescent females [63,71,73,75,77,81,85,92,94,95]. Programs targeting socioeconomically disadvantaged adolescents from low-development neighborhoods were conducted in 13 studies (38,2%) [62,63,65,74,75,79,85,87,89,91,92,93,97], with 6 of the 7 (85.7%) Australian studies conducted in low socioeconomic areas [79,85,87,89,91,93]. Quantitative studies were predominant (*n* = 18, 52.9%) [62,63,64,65,67,68,69,70,71,72,73,74,75,76,77,78,79,80], followed by mixed-methods (*n* = 10, 29.4%) [81,83,84,85,86,87,88,89,90,91] and qualitative studies (*n* = 6, 17.7%) [92,93,94,95,96,97]. Program duration ranged from 4 weeks to 4 years, with many programs lasting 12 weeks [70,82,83,85,87,90,93,94]. The most significant outcomes were observed in interventions lasting over three months [65,70,73,80,85,87,89,90,91,94] and exceeding one year [62,72,92,95,96,97] depending on the strategy employed. However, such extended durations occasionally affected participant engagement over time [95].

The most commonly used data collection instruments were as follows: questionnaires (*n* = 27, 79.4% [62,63,64,65,67,68,69,70,71,72,73,74,75,76,77,78,79,80,81,83,84,85,86,87,88,89,90]); interviews (*n* = 16, 47.0%) [75,81,83,84,85,86,87,88,89,90,91,92,93,94,95,96,97]; self-reports, including observations (*n* = 14, 41.2%) [62,67,71,72,76,77,78,80,83,85,88,90,92,97]; accelerometer (*n* = 9, 26.5%) [63,64,67,74,79,80,81,83,84]; and the use of measurement instruments (*n* = 5, 14.7%) [63,68,70,79,80], along with applications [67,89] and pedometers [84,89], to report baseline levels of PA. Outcome data included the use of applications, logbooks, and observations. The primary intervention assessment method was through self-reports and scale questionnaires after each action. Finally, outcomes of PA were presented in various forms, including different statistical variations, tests, analyses, movement frequency, and notes on levels of PA and energy expenditure.

### 3.3. Barriers to Participation in PA

**Figure 3:**
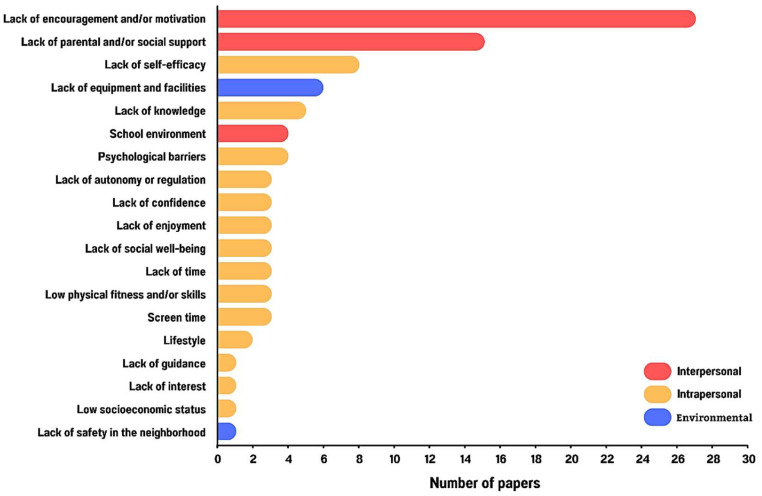
Barriers identified by intervention studies. Number of barriers identified in each intervention study, grouped into interpersonal, intrapersonal, and environmental categories.

Among the barriers identified in the studies involving adolescents participating in PA intervention programs, the following were notable (Figure 3):

1. Interpersonal, including social relationships, such as “lack of encouragement or motivation” (*n* = 23, 67.65%) [62,64,65,67,69,71,72,74,76,80,81,83,84,86,87,89,90,92,93,94,95,96,97] and “lack of parental and/or social support” (*n* = 13, 38.23%) [67,69,70,73,77,80,83,84,85,88,91,92,96].
2. Intrapersonal, including individual-related aspects, such as “lack of self-efficacy” (*n* = 8, 23.53%) [64,71,75,77,79,80,92,97], “psychological barriers” (*n* = 5, 14.70%) [64,73,78,84,97], “lack of social well-being” (*n* = 3, 8.82%) [78,83,84], “lack of enjoyment in physical activity” (*n* = 5, 14.70%) [63,71,73,79,85], “lack of knowledge” (*n* = 5, 14.70%) [65,77,78,86,87], “lack of autonomy or regulation” (*n* = 3, 8.82%) [79,95,97], “lack of time” (*n* = 3, 8.82%) [71,81,83], “lack of confidence” (*n* = 3, 8,82%) [69,85,90], “screen time” (*n* = 3, 8.82%) [62,64,88], “low physical fitness and/or skills” (*n* = 3, 8.82%) [63,68,97], “lifestyle” (*n* = 2, 5.88%) [65,86], “lack of guidance” (*n* = 1, 2.94%) [62], “lack of interest” (*n* = 1, 2.94%) [95], and “low socioeconomic status” (*n* = 1, 2.94%) [93] among adolescents.
3. Environmental, such as lack of adequate environments, are related to “lack of suitable equipment and facilities” (*n* = 6, 17.65%) [65,68,70,72,81,88], “school environment” (*n* = 4, 11.76%) [65,70,81,95], and “lack of safety in the neighborhood” (*n* = 1, 2.94%) [70].

### 3.4. Interventions to Address Barriers to Participation in PA

Among the barriers most frequently addressed when designing interventions for PA, the most frequently reported were “lack of motivation”, “lack of incentive”, and “lack of support” in the quantitative and qualitative studies (Figure 3).

Regarding the implementation of intervention programs, the following aspects were identified (Table 1 and Table 2):

1. Implementation Settings: Most studies (*n* = 31, 91.2%) [62,63,64,65,69,70,71,72,73,74,75,76,77,78,79,80,81,83,84,85,86,87,88,89,90,91,93,94,95,96,97] were conducted in or through schools.
2. Theoretical Frameworks: Some of the programs were explicitly based on theoretical frameworks, including cognitive, psychosocial, and socioenvironmental frameworks (*n* = 20, 47.0%) [62,63,64,67,69,70,71,79,80,81,83,84,85,86,87,88,89,90,91,92,93,94,95,96,97].
3. Intervention methods: Models for intervention in PA were implemented through various approaches, including workshops, practical activities, training sessions, lectures, classes, and achieving goals (*n* = 27, 79.41%) [62,63,64,65,69,70,71,72,73,74,75,76,77,78,79,80,81,85,87,89,91,92,93,94,95,96,97]. Recent studies incorporated educational materials [86] such as the use of brochures and provision of dedicated spaces and activities, including outdoor education [68]. Additional resources included activity kits with balls (basketballs, soccer balls, and volleyballs), jumping ropes, resistance bands, and activity sheets with promotional ideas for PA [88]. Some interventions used conversational agents with theory-based approaches through a channel (school websites or social media platforms) targeting students [90].
4. Electronic Equipment: A total of 16 interventions (47.0%) incorporated electronic devices for implementation or support, such as messages, brochures, and reminders throughout the duration of the program [62,67,69,70,76,79,80,81,84,85,86,87,89,90,93,94].

Following the analysis of the implementation of intervention programs, the studies were categorized into two large groups: 19 (55.9%) focused on “training, education, and theory” or educational approach [62,65,69,70,72,75,76,77,78,79,86,89,90,91,92,93,94,95,97]; and 10 (29.4%) emphasizing “participation in PA” or practical approach [67,68,74,75,82,83,84,85,87,88]. Eight studies (28.5%) incorporated both aspects, including achieving goals through applications based on theoretical information [63,64,71,73,80,81,87,96].

The interventions demonstrated significant effects on increasing PA, with several studies showing moderate (*n* = 7, 20.59%) [62,64,72,73,74,77,87] and high (*n* = 14, 47.06%) [63,65,70,71,75,76,80,82,91,92,94,95,96,97] effectiveness, particularly through enhanced motivation and encouragement among adolescents. Several studies (*n* = 13, 32.35%) failed to overcome all the barriers to implementation, achieving only partial effectiveness of the intervention (low) [67,69,78,79,83,84,85,89,93]. The effectiveness of each intervention can be found in Table 1 (final column), and for more details on the applied interventions, please refer to Supplementary Materials (File S2). However, a summary of the successful and ineffective strategies (Table 3), allowing for comparison and triangulation of the data, was prepared. Regarding the effectiveness of the intervention, the studies demonstrating the greatest success in reducing barriers to PA primarily addressed the psychosocial and/or psychological barriers (*n* = 10, 29.41%) [65,71,75,76,80,91,92,94,95,96,97], behavioral and/or socioenvironmental barriers (*n* = 5, 14.70%) [63,70,80,82,92], and other barriers (*n* = 3, 8.82%) [91,94,95], together with those employing mixed methods with an educational emphasis (Table 1). These findings underscore the importance of strategies that not only address barriers but also holistically engage participants to effectively promote PA.

**Table 3:** Intervention methods and classification of strategies.

Intervention Method	Ineffective Results	Inconsistent Results	Successful Results
“Cluster-RCT”: A total of 120 min/week of school-based PA, combining active learning (M1), physical education classes, and focused activities (M2).		[64]	
“Son la Pera”: Training adolescents to create challenges and implementing 10 social marketing activities to promote healthier choices.		[62]	
“Sport4FunO”: A total of 17 weekly sessions combining motor activities in school sports, optional post-school sports, and theoretical sessions (150 min/week).			[63]
“Jawbone UP MOVE”: Activity tracker and smartphone app used to set active minute goals and daily targets.	[67]		
“Strengthen Your Health”: Teacher training, health activities in the curriculum, PA opportunities, and health education for students and parents, with supervised PA during breaks.			[65,66]
“Outdoor Education Program”: Weekly 2-hour sessions with individual and team physical activities, including team building, navigation, climbing, archery, cycling, and hiking.	[68]		
“Move for Well-being in School”: (1) physical education classes, (2) classroom activities, (3) PA during breaks, and (4) thematic days.	[69]		
“Activ-O-Meter”: A web-based, tailored lifestyle education intervention for personalized advice.			[70]
(1) classroom PA, (2) health education sessions, and (3) online self-monitoring.			[71]
(1) teacher training workshops, (2) recreational breaks, and (3) access to sports equipment and spaces during recess.		[72]	
(1) lectures on the benefits of physical activity, (2) participant-chosen activities (aerobic dance, basketball, swimming, Tae Bo), and (3) self-monitoring, goal-setting, and problem-solving.		[73]	
(1) 60 min sessions twice a week, (2) 30 min of aerobic training, (3) 15 min of strength and flexibility training, and (4) 15 min of ball skills (soccer, netball).		[74]	
“Exercise Intervention Program”: (1) increased self-awareness and self-efficacy, (2) participation in various sports and physical activities twice a week, and (3) focus on skill mastery without judgment.			[75]
(1) lectures, group discussions, slides, videos, role-playing, and demonstrations, (2) competitions with parents, and (3) workshops on supporting children.			[76]
(1) 45–60 min group sessions (lectures, dramatizations, slides, reminder cards, tracking plans, and brochures) and (2) individual counseling and goal setting.		[77]	
“Health Oriented Pedagogical Project”: (1) active learning approach, (2) 45 min of extra daily PA, and (3) 225 additional minutes of PA per week.	[78]		
“RAW-PA”: (1) wearable activity tracker (Fitbit Flex), (2) digital resources via a private Facebook group, and (3) interactive missions with behavior change content.	[79]		
“Active by Choice Today”: (1) 60 min AFMV activities, (2) weekly behavioral and motivational skills component, and (3) based on Social Cognitive and Self-Determination Theory.			[80]
“Walking in Schools”: (1) 10–15 min of peer-led walking sessions, (2) reminder cards with tips and goal-setting, and (3) reward system for participation.			[81,82]
“GoActive”: (1) in- and out-of-school activities, (2) peer-led hierarchical leadership, (3) selection of two activities per week, and (4) focus on peer support, self-efficacy, self-esteem, and friendship quality.	[83]		
“The StepSmart Challenge”: (1) pedometer competition and (2) goal setting and monitoring.	[84]		
Sports program with new activities: (1) classroom yoga, pilates, and dance via instructional videos, (2) introductory tennis training, and (3) water games.	[85]		
Eight colored folders based on “On Your Feet Britain” and activities.	[86]		
“Raising Awareness of Physical Activity”: (1) activity tracking via electronic device and app, (2) weekly interactive goals, and (3) motivational videos and forums.		[87]	
(1) activity kits with sports equipment, (2) activity sheets for PA ideas, and (3) promotion of PA before and after school, individually or with family.	[88]		
“Active Teen Leaders Avoiding Screen Time”: (1) teacher training and seminars, (2) enhanced school sports and lunchtime PA sessions, (3) fitness equipment and pedometers, (4) parental strategies, and (5) smartphone apps and websites.	[89]		
Phyllis Conversational Agent: (1) theory-based support via school website or social media, (2) motivation and confidence modules, (3) barrier identification and personalized solutions, and (4) activity recommendations.	[90]		
“PA4E1”: (1) PE classes, (2) activity plans, (3) school sports, (4) PA during recess, (5) school PA policies, (6) community PA providers, and (7) parent communication.			[91]
“Girls Just Wanna Have Fun”: (1) workshops and awareness lectures and (2) sessions promoting self-control, effort, self-coaching, leadership, and transferability.			[92]
Through missions on a wearable activity tracker (Fitbit Flex) supported by digital materials delivered via social media (Facebook).	[93]		
“Intramural Program Planning”: (1) 25 student facilitators, (2) leadership training, (3) peer group planning, and (4) organization of engaging PA activities.			[94]
“Fit for Girls”: (1) PE teacher training and (2) school-specific action plans.			[95]
“Up2Us Sports SBYD”: (1) PE classes and school activities, (2) coaching and mentoring, (3) nutrition and PA sessions, and (4) coach training for inclusivity.			[96]
(1) integrated into PE classes, (2) self-control and participation, (3) group decision-making, (4) student-led activities, and (5) discussions on goal setting, life skills, and stress reduction.			[97]

Note: a sample size of <50 participants is insufficient for consistent results [98,99]. The table presents the classification of intervention strategies into three categories: ineffective (strategies that did not yield satisfactory results), inconsistent (strategies that showed results but lacked strong evidence), and successful (strategies that demonstrated positive and consistent outcomes).

### 3.5. Methodological Quality of Studies and Risk of Bias

Among the studies included in this systematic review on interventions and barriers of PA, 32 (94.1%) explicitly sought ethical approval, while only 20 (58.8%) declared no conflicts of interest.

The GRADE scores, which assessed the quality of evidence, classified 18 (52.9%) studies as “high quality”, 7 (20.6%) as “moderate quality”, 3 (8.8%) as “low quality”, and none as “very low quality”. For the qualitative studies assessed with CASP, all six (100%) demonstrated high methodological quality. The Downs and Black scale scores ranged from 52% to 93% (Table 4), with 26 (76.5%) achieving scores of 70% or higher, indicating a low risk of bias. The description of study quality and risk of bias is presented in Table 4.

## 4. Discussion

This systematic review synthesizes the findings of interventions aimed at reducing barriers to PA participation among adolescents. The included studies, conducted in 17 countries and involving more than 17,000 adolescents, evaluated programs addressing intrapersonal, interpersonal, and environmental barriers, including lack of time, insufficient knowledge, lack of parental support, absence of suitable environments, and inadequate facilities and equipment. The most effective interventions combined educational elements (e.g., theoretical instruction) with practical PA. Finally, most interventions were conducted in developed countries, with an emphasis on girls, reflecting efforts to reduce health inequalities.

A recent systematic review emphasized the importance of addressing psychological, emotional, cognitive, environmental, and sociocultural factors in adolescent interventions [13], corroborating our findings on programs that significantly minimized psychosocial, psychological, and behavioral barriers, thereby increasing PA participation. These findings align with previous studies [100], including systematics reviews and meta-analyses [28,100,101,102], reinforcing the role of social relationships in exercise motivation and in overcoming barriers that prevent adolescents from meeting PA recommendations [2,103,104]. However, there is no consistent evidence extending beyond movement-related behaviors, such as cognitive, academic, physical health, and/or psychological impacts [37]. The studies included in this review, which considered these barriers in program implementation, yielded significant results based on their proposals, most with a low risk of bias, providing compelling evidence of the effectiveness of these interventions. Conversely, a recent meta-analysis highlighted modest positive effects of interventions but also pointed to a high risk of bias in most analyzed studies [37], underscoring the importance of detailed reporting for replication. Additionally, promoting social relationships, such as peer support and influence, along with overcoming psychosocial barriers, emerges as an effective strategy to encourage PA in this group.

The most effective interventions were those combining theoretical foundations with practical implementation strategies, such as the “Active by Choice Today” program, which integrated “Social Cognitive Theory” and “Self-Determination Theory” to promote behavioral and socioenvironmental skills. This program included moderate-to-vigorous PA activities (60 min) chosen by students each week, along with a behavioral and motivational skills component (30 min) [80]. However, well-structured interventions, whether practical or theoretical, also demonstrated effectiveness in minimizing proposed barriers, emphasizing the need to assess the context, limitations of each proposal, and their replication. In this regard, interventions addressing multiple factors—including interpersonal, intrapersonal, and environmental—demonstrated greater efficacy and promising results [105,106,107], primarily due to their influence on behavioral change in this group [108,109]. Consequently, multifaceted PA interventions, incorporating various elements such as individuals, social and physical contexts, public policies, and others, have shown promising effects on behavioral change [13,107,110]. This aligns with recent reviews indicating that strategies combining multiple elements yield better health and quality-of-life outcomes compared to methods focused on a single-aspect approach [40,41], although the evidence base remains insufficient for definitive conclusions [38].

On the other hand, interventions focusing on interpersonal relationships require more time and adaptations to meet individual and cultural characteristics [77]. This highlights the need for tailored strategies that consider the environment and social relationships to promote sustainable behavioral changes among adolescent girls. In line with a review of the barriers and facilitators for improving the quality of primary healthcare in low- and middle-income countries with children and adolescents [111], the combination of individual and environmental changes is widely recognized, as effective behavioral changes require supportive policies and environments [40,112]. Thus, this integrated approach proves to be more effective, not only by informing participants about the scientific importance of PA but also by actively engaging them in practical experiences, encouraging lasting behavior change. Furthermore, interventions adopting approaches focused on goal modification, continuous monitoring, social support, and behavior and outcome management [27] demonstrated a more significant positive impact [28], reinforcing previous studies.

The results of this review, conducted with adolescents, corroborate the findings from the general population [13,113,114,115,116] and demonstrate the effectiveness of interventions in overcoming barriers to PA, such as time management re-education [117], encouragement and guidance on PA benefits, social support from friends and family, and the provision of adequate equipment and facilities, which, in turn, influence socioeconomic barriers [106]. Consistent with a previous review on school-based interventions aimed at increasing PA among adolescents [35], strategies such as PA sessions, educational resources, environmental modifications, peer support, and teacher training were widely adopted, leading to improved outcomes. Therefore, the influence of each barrier should be considered [13] to ensure that health promotion strategies and public policies encourage PA [114], given that the lack of information on the theoretical foundations of interventions and their diverse strategies hinders the identification of the most effective approaches [70]. Thus, this integrated approach proves to be more effective not only by informing participants about the scientific importance of PA but also by actively engaging them in practical experiences, fostering continued participation in PA.

The implementation of PA interventions for socioeconomically disadvantaged adolescents in schools is influenced by environmental barriers that should be prioritized [13]. In this regard, collaboration between schools, community partners, and authorities is essential to creating environments that facilitate PA participation, particularly in socioeconomically challenging contexts, through extracurricular programs and other initiatives that minimize access barriers [118,119]. Additionally, when implemented both in school and community settings, these initiatives can promote positive youth development, contribute to social and emotional learning [90], and address factors affecting overall well-being [120]. Thus, the findings of this review, indicating that more than half of the included studies were implemented in school settings, reinforce the importance of school-community partnerships in creating spaces that encourage PA, especially in socially vulnerable scenarios, through strategies such as integrating activities into the curriculum, extracurricular programs, and teacher training, as seen in many interventions with consistent results [37,121]. Consequently, as recommendations advance regarding implementation strategies in school and community settings—a widely supported measure in the literature [39]—these findings highlight the need for critical reviews on the effectiveness of adopted approaches and their adaptation to different contexts, both inside and outside school settings.

Moreover, the included studies tended to focus on interventions targeting female adolescents, with only one study exclusively on males, highlighting the scientific consistency of boys being more active [11]. Previous studies have reported higher rates of physical inactivity among adolescent girls [122,123,124] and identified more barriers to their regular PA participation [125,126,127], which may be related to this trend. Interpersonal factors, such as a lack of social support and gender-related cultural expectations, along with environmental barriers, such as the absence of safe and adequate spaces for PA, directly impact this group [10,14,128]. Gender-based sociocultural norms play a significant role, particularly in highly unequal contexts like Latin America, where safety and social environment are crucial determinants of adolescent health behaviors [128]. In line with a recent review emphasizing the need to reduce insufficient PA levels as part of global goals for 2030 [9], multisectoral efforts are essential to ensure the inclusion of all groups, preventing the widening of inequalities based on gender, age, or geographic location [12]. Notably, interventions with the most evident results lasted three months, while short-term interventions may be ineffective in modifying habits [86]. In some cases, longer interventions were considered excessive, affecting participant engagement over time [95].

The implementation of PA interventions was notably more prevalent in developed regions such as Australia, Europe, and the United States compared to low- and middle-income countries. However, in Australia, six of the seven studies (85.7%) were conducted in low socioeconomic neighborhoods [79,85,87,89,91,93]. This disparity may be linked to efforts to reduce social health inequalities, which appear to be increasing in developed countries [129]. Moreover, evidence, including a review of reviews, suggests a correlation between relative poverty and the health and well-being of children and adolescents in precarious conditions, even in wealthy countries, contributing to widening inequalities [130,131]. A previous study identified an intervention mismatch in wealthy countries compared to those with greater social vulnerability and population potential [132]. A previous study identified a discrepancy in intervention implementation between wealthy countries and those with greater social vulnerability and population potential [116]. Consequently, developed countries tend to implement more interventions to mitigate barriers than low- and middle-income countries [111], potentially enhancing the population-level impact of health promotion. Ultimately, concerning the use of accelerometers, pedometers, measurement instruments, and apps to report baseline PA levels, these methods were associated with less significant outcomes when used in isolation. This finding is supported by a recent meta-analysis that found no significant effects of interventions on total accumulated daily movement behaviors, as measured by accelerometers and changes in waist circumference [37].

Among the main methodological limitations of the analyzed studies, the frequent use of self-reported data to measure physical activity stands out, which may introduce memory and social desirability biases. Additionally, many studies have small sample sizes, making it difficult to generalize findings to broader populations. The lack of long-term follow-up compromises the assessment of intervention effectiveness, while non-random sampling may introduce systematic bias in participant selection. Another recurring issue is the lack of treatment fidelity control and the use of subjective measurements, which can affect the reliability of the results. These limitations highlight the need for caution when interpreting findings and reinforce the importance of future studies that address these methodological shortcomings.

This review highlighted several studies that contribute to the development and replication of effective interventions to increase PA. However, there are still opportunities to improve the design and reporting of these studies. The novelty of this review lies in compiling scientific evidence on interventions aimed at reducing barriers to participation in PA among adolescents. This compilation identified gaps in the literature and informed future studies. A notable strength is the use of rigorous, validated, and well-established methods, along with reviewer training. This systematic process enabled the exclusion of retracted studies.

Additionally, the limitations of the included studies, often overlooked in evaluations, were reported. However, this study also has limitations. Firstly, despite reviewer training, disagreements arose due to the subjective nature of the interventions addressing PA barriers, particularly in the initial review phase. This was largely due to the exclusion criteria that did not account for studies, identified as ineligible, only after a full-text review [133]. Second, the exclusion of gray literature in the review may have resulted in relevant information being missed. Methodological heterogeneity among the studies prevented a meta-analysis, as they differed in design, with similar barriers, both in terms of timing and methodology, and even in execution. Additionally, there was a lack of standardization in assessing intervention effectiveness. Future studies should apply rigorous methodologies, including bias control and context-sensitive approaches, ensuring durations longer than three months but under a year. Identifying barriers through targeted surveys and refining existing interventions, even those with inconsistent results, will help generate more reliable evidence.

Policymakers, educators, and healthcare professionals must evaluate the context and limitations of each intervention to ensure they meet adolescents’ specific needs. Future programs should minimize barriers and adapt strategies based on identified challenges, applying rigorous methodologies that integrate theoretical and practical approaches, bias control, and at least three months of interconnected actions. These efforts will generate reliable evidence to enhance intervention effectiveness and address physical inactivity among adolescents globally. Schools, particularly in underserved areas, can integrate PA into the curriculum through structured breaks and teacher training in inclusive methods. Programs like “Sport4Fun” conducted by Andruschko et al. [63] and “Walking In Schools” by Carlin et al. [81,82] effectively boost engagement by incorporating structured sessions and peer-led activities. Policymakers should enforce PA-friendly policies, allocate funding for structured programs like “Active by Choice Today” by Wilson et al. [80], and establish national guidelines promoting school-community collaboration, especially in low-income regions. The Outdoor Education Program by Chen et al. [68] has potential but requires larger sample sizes to confirm their effectiveness. In contrast, the program “Fit for Girls” by Mitchell et al. [95] has shown positive results in schools with gender-inclusion policies.

Community organizations can reinforce these efforts by hosting culturally relevant PA events, partnering with schools for affordable activities, and running awareness campaigns on the benefits of an active lifestyle. Ongoing monitoring and evaluation are crucial to track participation and health outcomes, ensuring interventions remain effective, sustainable, and adaptable to diverse adolescent needs.

## 5. Conclusions

The best results, with greater scientific rigor in some studies, were observed in addressing the psychosocial, psychological, and behavioral barriers, highlighting the importance of personalized approaches. The most effective interventions combined foundations with practical applications, as seen in adapted school programs and educational campaigns. However, the predominance of these interventions was conducted in developed countries, emphasizing the need for targeted efforts in low- and middle-income countries to promote PA equitably. These findings reinforce the need for critical reviews of the effectiveness of the approaches used and their adaptation to different contexts. Future studies should adopt rigorous methodologies, including bias control, long-term follow-ups, cost-effectiveness analyses, and the inclusion of gender-diverse populations, to generate more robust evidence. Finally, such evidence can support managers and the academic community in developing and implementing more effective interventions to promote PA among adolescents.

## Figures and Tables

**Table 4:** Methodological quality assessment and strength of evidence for quantitative and mixed studies.

		Conflict of Interests	Ethical Approval	Downs and Black Checklist																												GRADE
	Study (Year)			Section A										Section B			Section C							Section D						Total	Score^#^	
				1	2	3	4	5	6	7	8	9	10	11	12	13	14	15	16	17	18	19	20	21	22	23	24	25	26			
QUANTITATIVE (*n* = 18)																																
01	Aceves-Martins, M. et al., (2022) [62]	No	Yes	1	1	1	1	2	1	1	0	1	1	1	1	1	0	0	1	0	1	1	1	1	1	1	1	1	1	23/27	85%	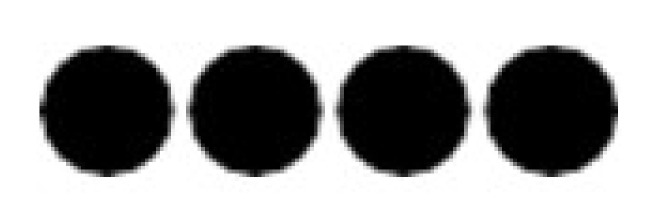
02	Andruschko, J. et al., (2018) [63]	–	Yes	1	1	1	1	2	1	1	0	0	1	1	1	1	0	0	0	1	1	1	1	1	1	1	1	1	1	22/27	81%	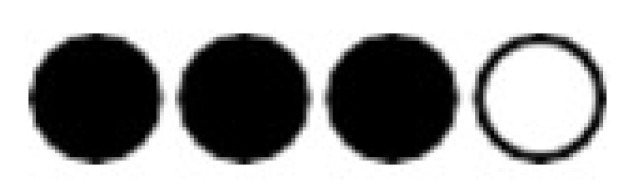
03	Åvitsland A. et al., (2020) [64]	No	Yes	1	1	1	1	2	1	1	0	1	1	1	1	1	0	1	1	1	1	1	1	1	1	1	1	1	1	25/27	93%	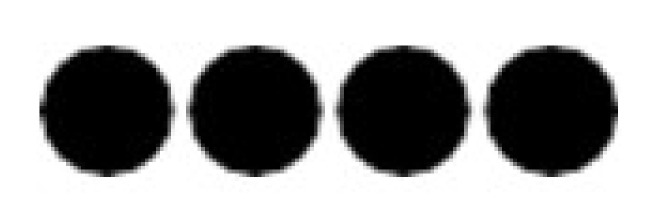
04	Barbosa Filho, V.C. et al., (2016) [65]	–	Yes	1	1	1	1	2	1	1	0	1	1	1	1	1	0	0	1	1	1	1	1	1	1	1	1	1	1	24/27	89%	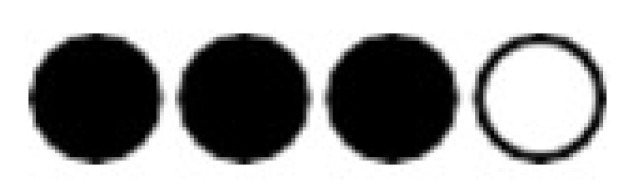
05	Bianchi-Hayes J. et al., (2018) [67]	–	Yes	1	1	1	1	2	1	1	0	1	1	1	1	1	0	0	1	1	1	1	1	1	1	1	1	1	1	24/27	89%	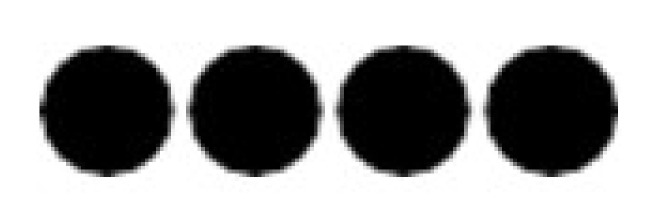
06	Chen, Y. et al., (2023) [68]	-	Yes	1	1	1	1	1	1	0	0	1	1	1	1	1	0	0	0	1	1	1	1	1	1	1	1	1	1	21/27	78%	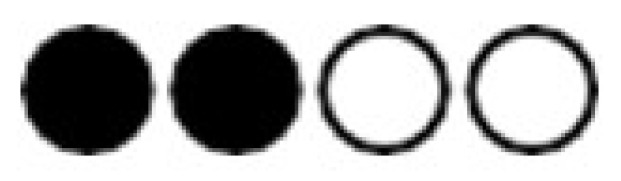
07	Christiansen, L.B. et al., (2018) [69]	–	Yes	1	1	1	1	2	1	1	0	1	1	1	1	0	1	0	1	1	1	1	1	1	1	1	1	1	1	24/27	89%	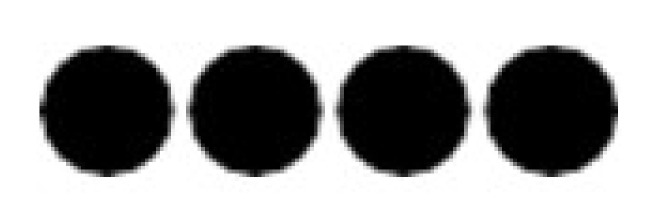
08	Cook, T.L. et al., (2014) [70]	No	Yes	1	1	1	1	0	1	1	0	0	1	1	1	0	0	0	1	1	1	1	1	1	1	1	1	1	1	20/27	74%	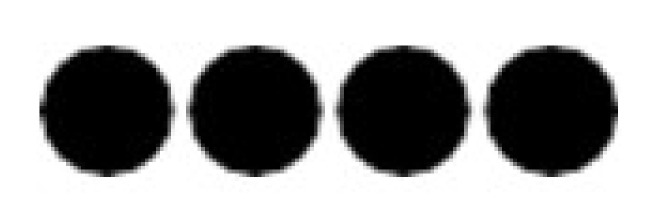
9	Dunton, G.F. et al., (2007) [71]	–	Yes	1	1	1	1	2	1	1	0	0	1	1	1	1	0	0	1	1	1	1	1	1	1	1	1	1	1	23/27	85%	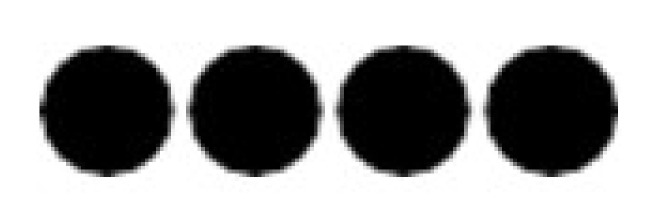
10	Gråstén, A. et al., (2015) [72]	–	Yes	1	1	1	1	2	1	1	0	0	1	1	1	1	0	0	1	0	1	1	1	1	1	0	0	1	0	19/27	70%	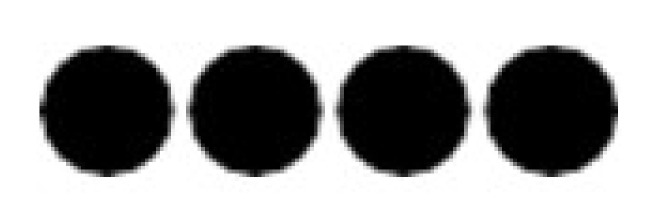
11	Jamner, M.S. et al., (2004) [73]	–	Yes	1	1	0	1	1	1	1	0	1	1	0	0	0	0	0	1	1	1	1	1	1	1	1	1	1	1	19/27	70%	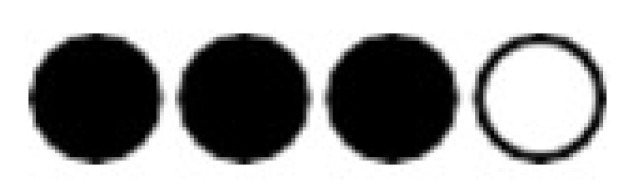
12	Lennox, A. et al., (2013) [74]	–	Yes	1	1	1	1	1	1	0	0	1	0	1	1	0	0	0	0	1	0	1	0	1	1	0	0	0	1	14/27	52%	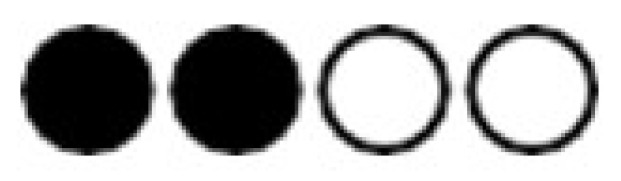
13	Lindgren, E.C. et al., (2011) [75]	–	Yes	1	1	1	1	1	1	1	0	1	1	0	0	0	0	0	1	1	1	1	1	1	1	1	1	1	1	20/27	74%	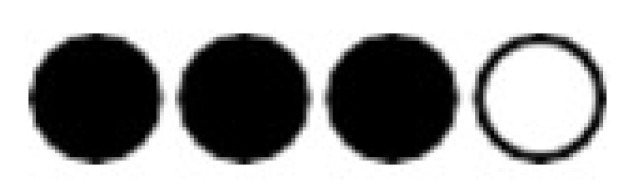
14	Sanaeinasab, H. et al., (2012) [76]	No	Yes	1	1	1	1	2	1	1	0	0	1	1	1	0	0	0	1	1	1	1	1	1	1	1	1	1	1	22/27	81%	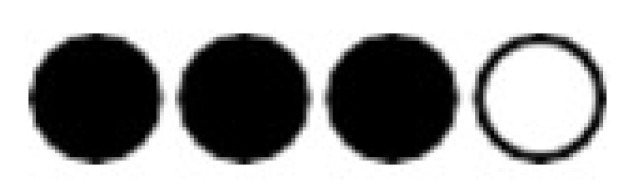
15	Taymoori, P. et al., (2008) [77]	No	Yes	1	1	1	1	1	1	1	0	1	1	1	1	0	1	0	1	1	1	1	1	1	1	1	1	1	1	23/27	85%	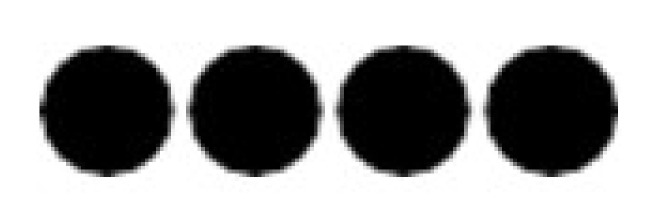
16	Tennfjord, M.K. et al., (2023) [78]	No	Yes	1	1	1	1	2	1	1	0	1	1	1	1	0	0	0	1	0	1	0	1	1	1	0	0	1	1	19/27	70%	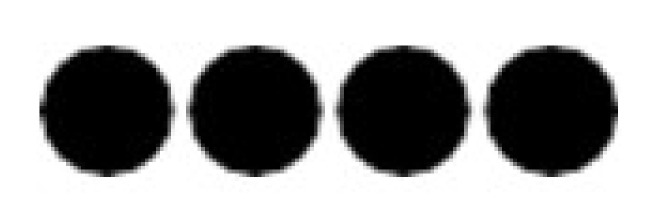
17	Verswijveren, S.J.J.M. et al., (2022) [79]	No	Yes	1	1	1	1	2	1	1	0	1	1	0	0	0	0	0	1	1	1	1	1	1	1	1	1	1	1	21/27	78%	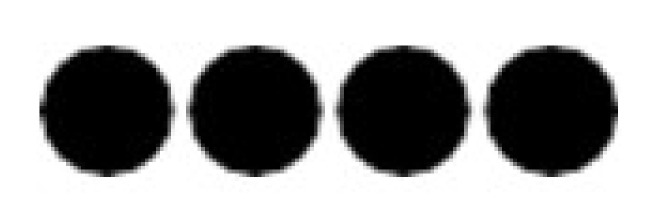
18	Wilson, D.K. et al., (2011) [80]	–	No	1	1	1	1	2	1	1	0	1	1	1	1	1	0	0	1	1	1	1	1	1	1	1	1	1	1	24/27	89%	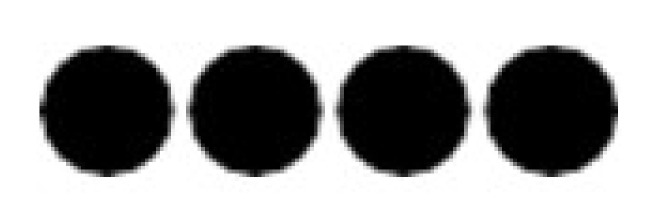
MIXED (*n* = 10)																																
19	Carlin, A. et al., (2018) [81]	No	Yes	1	1	1	1	2	1	1	0	0	1	1	1	0	0	0	1	1	1	0	1	1	1	1	1	1	1	21/27	78%	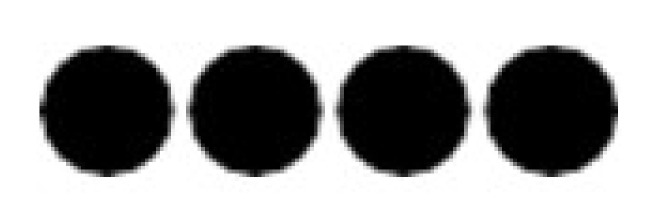
20	Corder, K. et al., (2020) [83]	No	Yes	1	1	1	1	2	1	1	1	1	1	1	1	1	0	0	1	1	1	1	1	1	1	1	1	1	1	25/27	93%	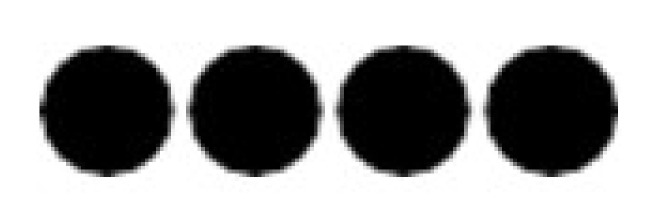
21	Corepal, R. et al., (2019) [84]	No	Yes	1	1	1	1	0	1	1	0	1	1	1	1	0	0	0	1	1	1	1	1	1	1	1	1	0	1	20/27	74%	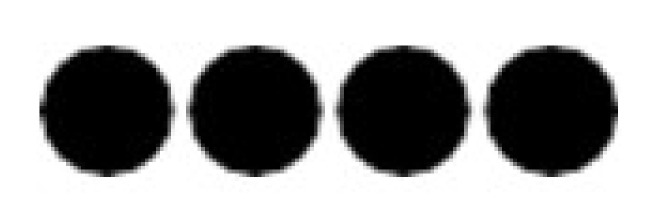
22	Dudley, D.A. et al., (2010) [85]	No	Yes	1	1	1	1	2	1	1	0	1	1	1	1	0	0	0	1	1	1	1	1	1	1	1	1	1	1	23/27	85%	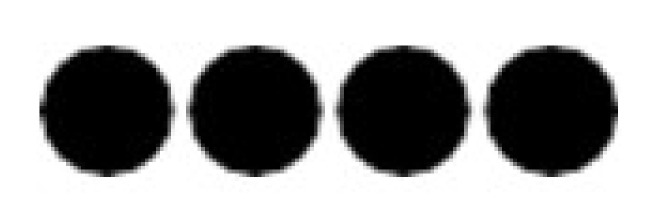
23	Ferreira Silva, R.M. et al., (2023) [86]	No	Yes	1	1	1	1	2	1	1	0	1	1	1	1	0	0	0	1	1	1	1	1	1	1	1	1	1	1	23/27	85%	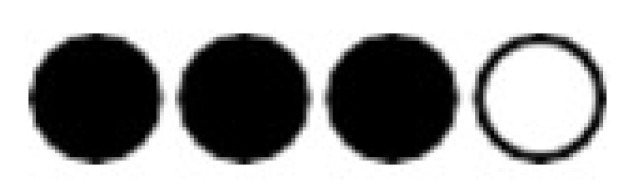
24	Koorts, H. et al., (2020) [87]	No	Yes	1	1	1	1	1	1	1	0	1	1	1	1	0	0	0	0	1	1	1	1	1	1	1	1	1	1	21/27	78%	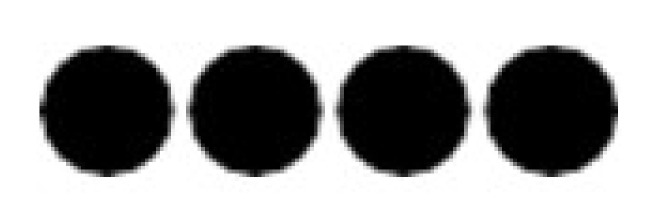
25	Kroshus, E. et al., (2023) [88]	No	Yes	1	1	1	1	2	1	1	0	1	1	1	1	0	0	0	1	1	1	1	1	1	1	1	1	1	1	23/27	85%	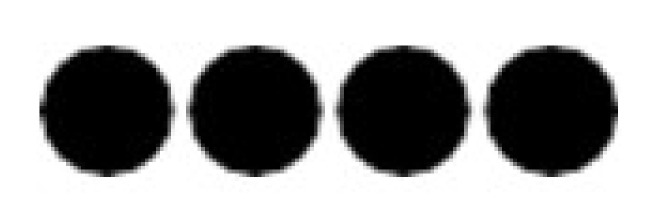
26	Lubans, D.R. et al., (2014) [89]	No	Yes	1	1	1	1	2	1	0	0	1	1	1	1	0	0	0	1	1	1	1	1	1	1	1	1	1	1	22/27	81%	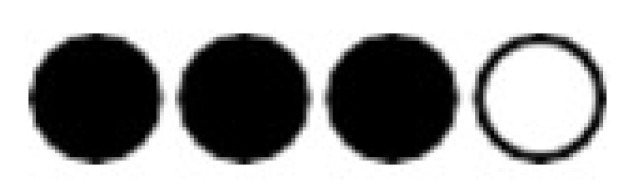
27	Moore, R. et al., (2024) [90]	No	Yes	1	1	1	0	1	0	1	0	1	0	1	1	0	0	0	0	1	1	1	1	1	1	1	1	0	1	17/27	63%	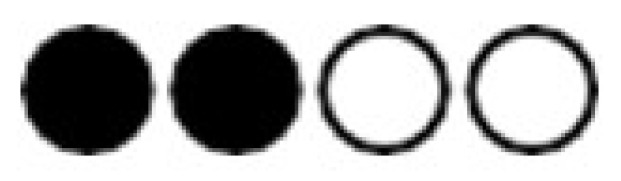
28	Sutherland, R. et al., (2020) [91]	No	Yes	1	1	1	1	2	1	1	0	1	1	1	1	0	0	0	1	1	1	1	1	1	1	1	1	1	1	23/27	85%	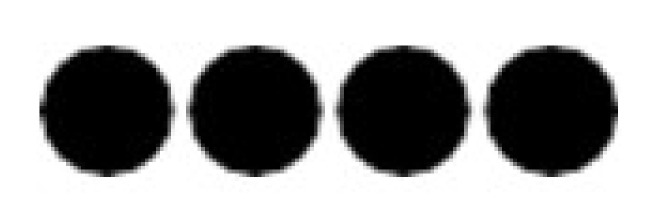
QUALITATIVE (*n* = 6)																																
29	Bean, C.N. et al., (2014) [92]	No	Yes																NA													☆ ☆ ☆
30	Drehlich, M. et al., (2020) [93]	No	Yes																NA													☆ ☆ ☆
31	Lodewyk, K.R. et al., (2023) [94]	-	Yes																NA													☆ ☆ ☆
32	Mitchell, F. et al., (2015) [95]	-	Yes																NA													☆ ☆ ☆
33	Pierre, S.T. et al., (2024) [96]	No	Yes																NA													☆ ☆ ☆
34	Wright, P.M. & Burton, S. (2008) [97]	-	No																NA													☆ ☆ ☆

## Data Availability

Not applicable.

## Supplementary Materials

The following supporting information can be downloaded at https://www.mdpi.com/article/10.3390/ijerph22060881/s1, File S1: Search strategy; File S2: Main version of the table used to extract data from the included studies; File S3: Checklist of the PRISMA 2020; Table S1: Keywords comprising the search strategy organized in blocks.
