# Supplementary material for: Interventions on Barriers to the Participation of Adolescents in Physical Activity: A Systematic Review
Source: Int J Environ Res Public Health. 2025 May 31;22(6):881. doi: 10.3390/ijerph22060881 (PMC12193246; doi:10.3390/ijerph22060881)
Supplement: Supplementary file 1 [file ijerph-22-00881-s001.zip › S1 File.pdf]

S1 File

Details of Boolean search string for each database.

Web of Science™ (WoS) Core Collection via native interface	
**Blocks and Returns**	**Search strings**
#1 (Return: ‹1,628,676›)	TS=("adolescent" OR "adolescents" OR "adolescence" OR "teen" OR "teens" OR "teenager" OR "teenagers" OR "youth" OR "young")
#2 (Return: ‹4,709,309›)	TS=("intervention" OR "interventions" OR "action" OR "actions" OR "program" OR "programs" OR "health education" OR "primary prevention" OR "health promotion" OR "primary health care")
#3 (Return: ‹3,628,451›)	TS=("barrier" OR "barriers" OR "obstacle" OR "obstacles" OR "challenge" OR "challenges" OR "difficulty" OR "difficulties" OR "facility access")
#4 (Return: ‹767,019›)	TS=("physical activity" OR "physical activities" OR "physical inactivity" OR "sedentary lifestyle" OR "sedentary behavior" OR "sedentary time" OR "exercise" OR "exercises")
#5 (Return: ‹3,963›)	(#1) AND (#2) AND (#3) AND (#4)
	**Search refinement**: 1. Document type: Article 2. Language: English **Return after refinement**: ‹3,928› studies in the test carried out in ‹01-08-2024›.

Scopus™ via native interface	
**Blocks and Returns**	**Search strings**
#1<br>(Return: ‹4,280,684›)	TITLE-ABS-KEY ("adolescent" OR "adolescents" OR "adolescence" OR "teen" OR "teens" OR "teenager" OR "teenagers" OR "youth" OR "young")
#2<br>(Return: ‹7,495,177›)	TITLE-ABS-KEY ("intervention" OR "interventions" OR "action" OR "actions" OR "program" OR "programs" OR "health education" OR "primary prevention" OR "health promotion" OR "primary health care")
#3<br>(Return: ‹4,942,588›)	TITLE-ABS-KEY ("barrier" OR "barriers" OR "obstacle" OR "obstacles" OR "challenge" OR "challenges" OR "difficulty" OR "difficulties" OR "facility access")
#4<br>(Return: ‹1,028,799›)	TITLE-ABS-KEY ("physical activity" OR "physical activities" OR "physical inactivity" OR "sedentary lifestyle" OR "sedentary behavior" OR "sedentary time" OR "exercise" OR "exercises")
#5<br>(Return: ‹5,547›)	(#1) AND (#2) AND (#3) AND (#4)
	**Search refinement**:<br>1. Document type: Article<br>2. Language: English<br>**Return after refinement**:<br>‹4,483› studies in the test carried out in ‹01-08-2024›.

MEDLINE/PubMed® via interface of the National Library of Medicine® (NLM®)	
**Blocks and Returns**	**Search strings**
#1<br>(Return: ‹1,005,498›)	"adolescent"[Title/Abstract] OR "adolescents"[Title/Abstract] OR "adolescence"[Title/Abstract] OR "teen"[Title/Abstract] OR "teens"[Title/Abstract] OR "teenager"[Title/Abstract] OR "teenagers"[Title/Abstract] OR "youth"[Title/Abstract] OR "young"[Title/Abstract]
#2<br>(Return: ‹3,115,571›)	"intervention"[Title/Abstract] OR "interventions"[Title/Abstract] OR "action"[Title/Abstract] OR "actions"[Title/Abstract] OR "program"[Title/Abstract] OR "programs"[Title/Abstract] OR "health education"[Title/Abstract] OR "primary prevention"[Title/Abstract] OR "health promotion"[Title/Abstract] OR "primary health care"[Title/Abstract]
#3<br>(Return: ‹1,709,782›)	"barrier"[Title/Abstract] OR "barriers"[Title/Abstract] OR "obstacle"[Title/Abstract] OR "obstacles"[Title/Abstract] OR "challenge"[Title/Abstract] OR "challenges"[Title/Abstract] OR "difficulty"[Title/Abstract] OR "difficulties"[Title/Abstract] OR "facility access"[Title/Abstract]
#4<br>(Return: ‹502,322›)	"physical activity"[Title/Abstract] OR "physical activities"[Title/Abstract] OR "physical inactivity"[Title/Abstract] OR "sedentary lifestyle"[Title/Abstract] OR "sedentary behavior"[Title/Abstract] OR "sedentary time"[Title/Abstract] OR "exercise"[Title/Abstract] OR "exercises"[Title/Abstract]
#5<br>(Return: ‹1,923›)	(#1) AND (#2) AND (#3) AND (#4)
	**Search refinement**:<br>1. Language: English<br>**Return after refinement**:<br>‹1,904› studies in the test carried out in ‹01-08-2024›.

Embase™ via native interface	
**Blocks and Returns**	**Search strings**
#1<br>(Return: ‹1,295,339›)	'adolescent':ti,ab,kw OR 'adolescents':ti,ab,kw OR 'adolescence':ti,ab,kw OR 'teen':ti,ab,kw OR 'teens':ti,ab,kw OR 'teenager':ti,ab,kw OR 'teenagers':ti,ab,kw OR 'youth':ti,ab,kw OR 'young':ti,ab,kw
#2<br>(Return: ‹4,065,528›)	'intervention':ti,ab,kw OR 'interventions':ti,ab,kw OR 'action':ti,ab,kw OR 'actions':ti,ab,kw OR 'program':ti,ab,kw OR 'programs':ti,ab,kw OR 'health education':ti,ab,kw OR 'primary prevention':ti,ab,kw OR 'health promotion':ti,ab,kw OR 'primary health care':ti,ab,kw
#3<br>(Return: ‹2,142,371›)	'barrier':ti,ab,kw OR 'barriers':ti,ab,kw OR 'obstacle':ti,ab,kw OR 'obstacles':ti,ab,kw OR 'challenge':ti,ab,kw OR 'challenges':ti,ab,kw OR 'difficulty':ti,ab,kw OR 'difficulties':ti,ab,kw OR 'facility access':ti,ab,kw
#4<br>(Return: ‹680,005›)	'physical activity':ti,ab,kw OR 'physical activities':ti,ab,kw OR 'physical inactivity':ti,ab,kw OR 'sedentary lifestyle':ti,ab,kw OR 'sedentary behavior':ti,ab,kw OR 'sedentary time':ti,ab,kw OR 'exercise':ti,ab,kw OR 'exercises':ti,ab,kw
#5<br>(Return: ‹2,709›)	(#1) AND (#2) AND (#3) AND (#4)
	**Search refinement**:<br>1. Language: English<br>**Return after refinement**:<br>‹1,951› studies in the test carried out in ‹01-08-2024›.

SPORTDiscus® Plus via EBSCOhost™ interface	
**Blocks and Returns**	**Search strings**
#1<br>(Return: ‹124,396›)	TI ("adolescent" OR "adolescents" OR "adolescence" OR "teen" OR "teens" OR "teenager" OR "teenagers" OR "youth" OR "young") OR AB ("adolescent" OR "adolescents" OR "adolescence" OR "teen" OR "teens" OR "teenager" OR "teenagers" OR "youth" OR "young") OR KW ("adolescent" OR "adolescents" OR "adolescence" OR "teen" OR "teens" OR "teenager" OR "teenagers" OR "youth" OR "young")
#2<br>(Return: ‹245,951›)	TI ("intervention" OR "interventions" OR "action" OR "actions" OR "program" OR "programs" OR "health education" OR "primary prevention" OR "health promotion" OR "primary health care") OR AB ("intervention" OR "interventions" OR "action" OR "actions" OR "program" OR "programs" OR "health education" OR "primary prevention" OR "health promotion" OR "primary health care") OR KW ("intervention" OR "interventions" OR "action" OR "actions" OR "program" OR "programs" OR "health education" OR "primary prevention" OR "health promotion" OR "primary health care")
#3<br>(Return: ‹83,299›)	TI ("barrier" OR "barriers" OR "obstacle" OR "obstacles" OR "challenge" OR "challenges" OR "difficulty" OR "difficulties" OR "facility access") OR AB ("barrier" OR "barriers" OR "obstacle" OR "obstacles" OR "challenge" OR "challenges" OR "difficulty" OR "difficulties" OR "facility access") OR KW ("barrier" OR "barriers" OR "obstacle" OR "obstacles" OR "challenge" OR "challenges" OR "difficulty" OR "difficulties" OR "facility access")
#4<br>(Return: ‹236,243›)	TI ("physical activity" OR "physical activities" OR "physical inactivity" OR "sedentary lifestyle" OR "sedentary behavior" OR "sedentary time" OR "exercise" OR "exercises") OR AB ("physical activity" OR "physical activities" OR "physical inactivity" OR "sedentary lifestyle" OR "sedentary behavior" OR "sedentary time" OR "exercise" OR "exercises") OR KW ("physical activity" OR "physical activities" OR "physical inactivity" OR "sedentary lifestyle" OR "sedentary behavior" OR "sedentary time" OR "exercise" OR "exercises")
#5<br>(Return: ‹581›)	(#1) AND (#2) AND (#3) AND (#4)
	**Search refinement**:<br>1. Document type: Article<br>2. Language: English<br>**Return after refinement**:<br>‹559› studies in the test carried out in ‹01-08-2024›.

Note: CINAHL is acronymous of Cumulative Index to Nursing and Allied Health Literature®; and EBSCO is acronymous of Elton Bryson Stephens Company.
