# Supplementary material for: Interventions on Barriers to the Participation of Adolescents in Physical Activity: A Systematic Review
Source: Int J Environ Res Public Health. 2025 May 31;22(6):881. doi: 10.3390/ijerph22060881 (PMC12193246; doi:10.3390/ijerph22060881)
Supplement: Supplementary file 1 [file ijerph-22-00881-s001.zip › S1 Table.pdf]

S1 Table: Keywords comprising the search strategy organized in blocks

Blocks (*PICO*)	Keywords
1<br>*P*	“adolescent” OR “adolescents” OR “adolescence” OR “teen” OR “teens” OR “teenager” OR “teenagers” OR “youth” OR “young”
2<br>*I*	“intervention” OR “interventions” OR “action” OR “actions” OR “program” OR “programs” OR “health education” OR “primary prevention” OR “health promotion” OR “primary health care”
3<br>*C*	“barrier” OR “barriers” OR “obstacle” OR “obstacles” OR “challenge” OR “challenges” OR “difficulty” OR “difficulties” OR “facility access”
4<br>*O*	“physical activity” OR “physical activities” OR “physical inactivity” OR “sedentary lifestyle” OR “sedentary behavior” OR “sedentary time” OR “exercise” OR “exercises”
**Search *string***:	(1) AND (2) AND (3) AND (4)

Note: PICO stands for Population, Intervention, Comparison, Outcome; furthermore, it is a framework created to define the issue addressed by the systematic review.
