# Supplementary material for: Interventions on Barriers to the Participation of Adolescents in Physical Activity: A Systematic Review
Source: Int J Environ Res Public Health. 2025 May 31;22(6):881. doi: 10.3390/ijerph22060881 (PMC12193246; doi:10.3390/ijerph22060881)
Supplement: Supplementary file 1 [file ijerph-22-00881-s001.zip › S2 File 02.04.25.pdf]

S2 File

Main version of the table used to extract data from the included studies.

#	AUTHOR, YEAR, AND PLACE OR COUNTRY OF STUDY	BARRIERS	INTERVENTION USED AND TIME	SAMPLE	PERIOD IN WHICH DATA COLLECTION OCCURRED	INSTRUMENT USED FOR DATA COLLECTION AND TYPE OF ANALYSIS PERFORMED	MAIN RESULTS
			**QUANTITATIVE**				
**1**	Aceves-Martins, M. *et al*. (2022).<br><br>Spain	Lack of encouragement, guidance, screen time	**INTERVENTION:**<br>"Son la Pera": (1) training five adolescents per country to create challenges and (2) designing and implementing 10 activities using social marketing (SM) to promote healthier choices.<br><br>**TIME:** 12 months	169 (13–16 years old)	2014-2015	**INSTRUMENT:**<br>Self-reported Questionnaire.<br><br>**ANALYSES:**<br>Logistic regression, linear models, and McNemar tests.	The intervention improved weekly participation in PA (versus the control group, p=0.047), particularly among males (p=0.050) attending high schools in low-income neighborhoods.<br><br>There were no improvements in sedentary behavior, measured as daily screen time.
**2**	Andruschko, J. *et al*. (2018).<br><br>Australia	Low physical fitness and lack of enjoyment	**INTERVENTION:**<br>Sport4Fun Program: 1) 17 weekly sessions of motor activities (sprint running, catching, throwing, two-handed striking, and kicking) for 90 minutes during mandatory school sports; 2) 60-minute post-school sports activities (optional); 3) a theoretical component (three 15-minute sessions) during classroom time each week.<br><br>**TIME:** 6 months	292 girls (from 7th to 9th grade)	2008	**INSTRUMENT:**<br>Scales: "Harter's Perceived Competence Scale for Children" and "Enjoyment of Physical Activity".<br><br>Questionnaire and accelerometers.<br><br>**ANALYSES:**<br>One-way analysis of covariance.	The intervention led to a slight increase in perceived physical competence and enjoyment among girls in the intervention group, but these increases were greater in the control group, resulting in a significant difference between groups (P=0.01), with a large effect size for physical competence (D=–1.02) and a small-to-medium effect size for enjoyment of physical activity (D=0.47) in favor of the control group.
**3**	Åvitsland A. *et al.* (2020).<br><br>Norway	Lack of motivation, self-efficacy, and psychosocial mechanism	**INTERVENTION:**<br>Intervention "Cluster-RCT" based on different theoretical frameworks aiming to increase physical activity in school by approximately 120 minutes per week.<br><br>The "Active Learning" intervention model (M1) consisted of 30 minutes of physically active learning, 30 minutes of physical	1,391 (14–15 years old)	2020	**INSTRUMENT:**<br>Questionnaire "Strengths and Difficulties" e Accelerometry.<br><br>**ANALYSIS:**<br>Models Linear mixed and IBM SPSS Statistics 25.	The M1 intervention model had a total difficulty score (TDS) 6% higher (b=0.6; 95% CI=0.003–1.17; p=0.049) compared to the control, and M2 had a 3.4% lower SES (–0.2 to 0.0; p=0.039) than the control.<br><br>During the school period, M1 had 13% fewer steps per minute (b=−65.0; 95% CI= −90.2 to −39.8; p<0.001), 6% more sedentary time (b=14.2; 95% CI=10.2–18.2; p<0.001), and 14% less moderate-to-vigorous physical

			activity, and a 60-minute physical education class.<br><br>The “Don’t Worry, Be Happy” intervention model (M2) consisted of a physical education class and a physical activity class, both focused on facilitating students' interest, responsibility, and social relationships.<br><br>**TIME:** 29 weeks				activity (*b*=−4.3; 95% CI= −5.9 to −2.6; *p*<0.001) compared to the control.<br><br>M1 showed favorable results in the subgroup with higher levels of psychological difficulties at the start of the study (b = −2.9; −5.73 to −0.07; p = 0.045) and in immigrants (b = −1.6; −3.53 to 0.27; p = 0.093).<br>The results indicated beneficial effects in two subgroups: those with higher baseline levels of psychological difficulties and immigrants.
**4**	Barbosa Filho, V. C. *et al*. (2016); and<br>** Barbosa Filho, V. C. *et al*. (2019).<br><br>Brazil	Lack of knowledge and encouragement, screen time, school environment, lack of equipment, and lifestyle	**INTERVENTION:**<br>Program "Strengthen Your Health": teacher training, health-related activities in the curriculum, active opportunities in the school environment (availability of equipment for PA), and health education for students and parents. Additionally, supervised sessions and encouragement for PA during breaks.<br><br>**TIME:** 4 months	1,085 (11–18 years old) students from 6 schools	2014	**INSTRUMENT:**<br>Questionnaire.<br><br>**ANALYSES:**<br>Linear models and binary logistic regression, linear models, McNemar tests, independent *t*-test, and chi-square test.	The intervention resulted in an increase in the number of adolescents meeting physical activity guidelines (5.3%; 95% CI=0.8–9.8); an increase in computer use for <2 hours per day (8.6%; 95% CI=3.8–13.4); and a higher likelihood of engaging in physical activity at recommended levels (OR=1.44; 95% CI=1.00–2.08).<br><br>Thus, there was an increase in weekly moderate-to-vigorous PA (effect size = 0.17), participation in popular games (effect size = 0.35), and the amount of PA per week (effect size = 0.27) among students (all *P*<0.05).
**5**	Bianchi-Hayes J. *et al*. (2018).<br><br>USA	Lack of motivation and parental support	**INTERVENTION:**<br>Jawbone UP MOVE activity tracker and its smartphone app to reflect recommendations for active minutes goals, serving as a motivation tool with daily targets.<br><br>**TIME:** 10 weeks	9 (14–16 years old) overweight or obese	2013	**INSTRUMENT:**<br>Self-reported questionnaire and smartphone app data from the activity tracker.<br><br>**ANALYSES:**<br>Descriptive statistics, frequencies, percentages for categorical variables, mean (SD) for continuous variables, and Spearman correlations.	The intervention group achieved step goals at least one-third of the time (35% and 39%) and active minute goals more than half of the time (55% and 83%).<br><br>Weekly step counts and active minutes between parents and adolescents were significantly correlated (step count: *r*=0.36, adjusted *P*=0.002; active minutes: *r*=0.30, *P*=0.007).
**6**	Chen, Y. et *al*., (2023).<br><br>USA	Lack of suitable environments and low physical	**INTERVENTION:**<br>Outdoor Education Program (OEP): weekly two-hour sessions including physical activities requiring individual skills and team coordination. Examples of	15 students (12–17 years old)	2023	**INSTRUMENT:**<br>Questionnaire and Fitness Test (PACER, standard and 90° push-up tests, back-saver sit and reach test).	The intervention led to improvements in two components of physical fitness: VO2max (t(14)=–3.40, p<0.05, d=0.88) and the number of push-ups performed (t(14)=–3.03, p<0.05, d=0.78). Seven out of the 15 participants met Fitnessgram standards for

		fitness and skills	activities: a) team building, b) navigation – trail walking, c) orienteering, d) climbing and basic/advanced rappelling, e) archery, f) cycling, and g) advanced hiking.<br><br>**TIME:** 9 weeks			**ANALYSES:**<br>Paired-samples *t*-tests and Mann-Whitney *U*-tests.	three or more components at the pre-test, and six of these participants maintained those standards at the post-test.<br><br>Additionally, the program had a more significant impact on male participants (mean rank = 9.38, 9.25, and 9.21), with all significant differences in the post-test favoring males over females.
**7**	Christiansen, L.B. *et al.* (2018).<br><br>Denmark	Lack of self-confidence, lack of motivation and support	**INTERVENTION:**<br>The "Move for Well-being in School" program focused on addressing the needs for competence, autonomy, and relatedness to enhance intrinsic motivation for physical activity. It incorporated workshops, training courses, educational materials, planning guides, and physical education lesson plans to integrate physical activity throughout the school day. The program included: 1) physical education classes, 2) classroom activities, 3) physical activities during breaks, and 4) thematic days.<br><br>**TIME:** 9 months	2,797<br>(10–13 years old)	2015–2016	**INSTRUMENT:**<br>Self-scale questionnaires.<br><br>**ANALYSES:**<br>Mixed linear models, correlation coefficients, test-retest, and likelihood ratio tests.	The intervention and the activities provided were not effective in improving physical self-perception. However, self-perception of sports competence showed an intervention effect of 0.03 (95% CI= –0.01 to 0.08), and global self-esteem had the lowest effect at 0.00 (–0.046 to 0.051). For physical self-esteem, the increase was 0.11 in the intervention group and 0.09 in the control group, with an intervention effect of 0.02 (95% confidence interval; CI= –0.03 to 0.07). The greatest intervention effect was observed for self-perceived sports competence (coefficient = 0.03, 95% CI = –0.01 to 0.08), although it was not statistically significant.
**8**	Cook, T. L. *et al.* (2014).<br><br>Europe	Neighborhood safety, sports facilities in the neighborhood, lack of sports facilities at school, social support	**INTERVENTION:**<br>"Activ-O-Meter", an advice Web-based tailored Lifestyle Education Intervention.<br><br>**TIME:** 3 months	536 students<br>(12–17 years old)	2014	**INSTRUMENT:**<br>Questionares<br>PA: IPAQ-A.<br><br>Barriers, Social support, and others: 11 questionnaire items, with 3-point Likert-type scales.<br><br>**ANALYSES:**<br>Linear regression analysis.	PEB regarding neighborhood safety mediated the effect of the intervention on cycling for transportation (*ab*=22.266, 95% CI=3.898–55.505, %ME=54.3), walking for transportation (*ab*=34.798, 95% CI=1.954–71.764, %ME >100), walking in leisure time (*ab*=28.177, 95% CI=5.247–66.146, %ME=95.5), MPA in leisure time (*ab*=45.872, 95% CI=6.898–83.593, %ME=55.0), VPA in leisure time (*ab*=22.978, 95% CI=3.380–60.021, %ME=47.2), and MVPA (*ab*=14.831, 95% CI=9.714–24.568, %ME=16.6). Social support from a sports partner suppressed the effect of the intervention on VPA in leisure time (*ab*= –10.807, 95% CI=–30.240 to –1.712) and MVPA (*ab*=–14.706, 95% CI=–27.038 to –9.492).

**9**	Dunton, G. F. *et al.* (2007).<br><br>California	Lack of self-efficacy, lack of motivation, time, and enjoyment	**INTERVENTION:**<br>Supervised physical activity in the classroom, health education, and internet-based self-monitoring. Intervention classes were conducted five days a week for 60 minutes per day (approximately 40 minutes of activity time), with one day per week dedicated to an educational component. Class activities included yoga, aerobics, basketball, swimming, strength training, hip-hop dancing, soccer, walking, and kickboxing. Weekly lectures and discussions covered topics such as time management, body image, motivation, nutrition, and strength training.<br><br>**TIME:** 9 months	122 girls (14–17 years old)	2005	**INSTRUMENT:**<br>Recall, cycle ergometer exercise test, and self-report questionnaire.<br><br>**ANALYSES:**<br>Multilevel random coefficients modeling, regression modeling, mediation analysis, and missing data treatment.	Participants in the intervention experienced improvements in vigorous physical activity (p=0.001) and cardiovascular fitness (p=0.008). The intervention also had a significant effect on internal (p=0.025) and external (p=0.006) barriers to physical activity, although the effect was not in the hypothesized direction.<br><br>There were positive changes in global self-efficacy (p=0.022) and exercise enjoyment (p = 0.026), which were associated with improvements in cardiovascular fitness after accounting for changes in other psychosocial variables included in the model.
**10**	Gråstén, A. *et al.* (2015).<br><br>Finland	Lack of motivation and lack of access to environments and equipment.	**INTERVENTION:**<br>Teacher training workshops on promoting physical activity, recreational activities as controlled breaks, and access to spaces and equipment for sports, games, and activities during recess.<br><br>**TIME:** 12 months	847 (12–14 years old)	2011–2012	**INSTRUMENT:**<br>Self-Report Questionnaires: ("Motivation Climate in Physical Education Scale," "Perception of Success Questionnaire," "Health Behavior in School-aged Children Research Protocol").<br><br>**ANALYSES:**<br>Descriptive analyses, independent t-tests, analysis of covariance (ANCOVA), and regression analysis.	The intervention had a moderate effect on self-reported MVPA (β=0.19). A strategic model was implemented to prevent declining levels of MVPA participation, resulting in higher levels of MVPA (t[764]=2.77, p<0.01, d=0.20). The control group exhibited higher levels of ego-involving climate (T0: t[754]=40.06, p<0.001, d=0.30; T1: t[834]=2.93, p=0.004, d=0.20), ego orientation (t[845]=2.72, p<0.001, d=0.19), and MVPA (t[764]=2.77, p<0.01, d=0.20).
**11**	Jamner, M. S. *et al.* (2004).<br><br>California	Psychosocial factors, lack of social support, and lack of enjoyment when participating in PA	**INTERVENTION:**<br>The program included lectures and discussions focused on the benefits of physical activity. Additionally, participants engaged in activities (aerobic dance, basketball, swimming, Tae Bo, and others) of their choice five times a week for 60 minutes. Strategies for behavior	58 sedentary girls	2004	**INSTRUMENT:**<br>Questionnaires ("Physical Fitness and Physical Activity" and "Psychosocial Assessments") and scales.<br><br>**ANALYSES:**	The intervention had an effect on cardiovascular fitness (F=6.23, p=0.017) and lifestyle (F(group × time)=9.025, p=0.005). Family support was positively correlated with both lifestyle activities (r=0.483, p<0.05). Overall internal self-efficacy decreased overtime, from 2.87 (SD=0.71) at the start of the study to 2.67 (SD=0.51) in 4 months. In contrast, the overall enjoyment of activities increased from 3.51 (SD=0.62) at the beginning of the study to 3.77 (SD=0.62) in 4

			change included self-monitoring, goal-setting, and problem-solving.<br><br>**TIME:** 4 months			Repeated measures analysis of variance, *t*-tests, and logistic regression.	months. There was no effect of the intervention on psychosocial factors related to exercise.
**12**	Lennox, A. *et al*. (2013).<br><br>South Africa	Lack of incentive	**INTERVENTION:**<br>Physical activity based in schools was conducted twice a week, with a duration of 60 minutes per session after school hours. The program was divided into 30 minutes of aerobic training, 15 minutes of strength and flexibility training, and 15 minutes of sports activities related to ball skills. The sessions began with aerobic training (aerobic exercises, dance, boxing Kata), after which participants were divided into two smaller groups, where they engaged in strength and flexibility exercises and specific ball skills sessions (soccer and netball).<br><br>**TIME:** 6 months	252 (aged 14.8 years)	2012	**INSTRUMENT:**<br>Socioeconomic questionnaire and accelerometers<br><br>**ANALYSES:**<br>Post hoc analysis and Bleep test. Descriptive statistics of means, standard deviations (*SD*), and maximum and minimum values.	The results reveal that the experimental subgroup with higher frequency in the program (>70%) showed higher levels of aerobic fitness and physical activity. Regarding average energy expenditure during the intervention, the experimental group spent, on average, 58.5% of the 54.7 minutes in moderate activities and 14.8% in vigorously intense activities, with an average of 40.1 minutes of the 54.7 minutes in moderate to intense activities.
**13**	Lindgren, E. C. *et al*. (2011).<br><br>Sweden	Lack of self-efficacy	**INTERVENTION:**<br>The Exercise Intervention Program (EIP) was based on empowerment and had a set duration. Overall, it aimed to increase participants' awareness of their own interests and needs, allowing them to play an active role in the development of the program. Another goal of the empowerment process was to strengthen the participants' perceived self-efficacy. They were invited to participate in various sports and physical activities twice a week, during which they were able to learn to master the activities they selected without feeling embarrassed about their bodies or skill level.<br><br>**TIME:** 6 months	110 girls (13–19 years old)	2002–2003	**INSTRUMENT:**<br>Questionnaires ("General Self-Efficacy Scale"; "Support and Social Barriers to Exercise Self-efficacy Questionnaire").<br><br>**ANALYSES:**<br>Mann-Whitney *U*-test and Wilcoxon signed-rank test for paired samples.	The girls in the intervention group increased their perceived general self-efficacy (p=0.004). Both groups showed an increase in physical fitness levels (intervention, p=0.06 and control, p=0.013). There was an increase in physical fitness levels (intervention, p=0.06 and control, p=0.013). The BMI in the intervention group was maintained, while the BMI increased in the control group (p=0.031).

**14**	Sanaeinasab, H. *et al.* (2012). Iran	Lack of encouragement and support	**INTERVENTION:** Educational Program with interactive sessions (1–1.5 hours each), which included lectures, group focus discussions, slide and video presentations, role-playing, and demonstrations. Additionally, there were competitions with the parents and a workshop with tips on how parents could support their children. **TIME:** 2 months	165 (13–15 years old)	2010–2011	**INSTRUMENT:** Self-reported questionnaires based on HPM constructs ("benefits and barriers of action"; "self-efficacy"; "activity-related affect"; "interpersonal influences"; and "situational influences"). **ANALYSES:** Multiple regression analysis, *t*-test, chi-square test, and Pearson correlation test.	The intervention showed that the benefits of physical activity, the social support component of interpersonal influences, and situational influences correlated with weekly physical activity time with $p<0.05$. Weekly physical activity time increased from 131.93±38.68 to 264.62±72.61 among participants in the intervention group (considered significant). A significant difference in weekly physical activity was found between girls and boys ($p<0.001$) in both the intervention and control groups.
**15**	Taymoori, P. *et al.* (2008). Iran	Lack of support, knowledge, and self-efficacy	**INTERVENTION:** Group educational sessions of 45 to 60 minutes (at the start of the study, 4th, 10th, and 18th week) with lectures, dramatizations, slides, reminder cards, physical activity tracking plans, and educational brochures. Additionally, individual counseling and personal physical activity goal setting were included. The intervention was based on Pender's Health Promotion Model (HP), integrating the Health Promotion Model with selected constructs from the Transtheoretical Model (THP), and included a control group (CON). **TIME:** 6 months	161 girls (9th and 10th grade)	2007–2008	**INSTRUMENT:** Self-reported scale questionnaire. **ANALYSES:** Post hoc test and univariate analysis of covariance.	The intervention increased the mean physical activity from baseline to post-intervention ($p<0.0001$) and at the start of follow-up ($p<0.0001$). Significant differences were found for counterconditioning, $F=11.97$, $p=0.000$, $\eta^2=0.16$; stimulus control, $F=14.82$, $p=0.000$, $\eta^2=0.15$; total physical activity minutes per week, $F=31.50$, $p=0.000$, $\eta^2=0.29$; and average physical activity minutes per day, $F=39.94$, $p=0.000$, $\eta^2=0.34$. Differences were also observed for counterconditioning, $F=7.83$, $p=0.001$; stimulus control, $F=14.02$, $p=0.04$; total physical activity minutes per week, $F=4.32$, $p=0.01$; and average physical activity minutes per day, $F=5.0$, $p=0.008$.
**16**	Tennfjord, M. K. *et al.* (2023). Norway	Psychosocial problems (increased well-being and self-concept)	**INTERVENTION:** "The Health Oriented Pedagogical Project": Focused on shifting from passive learning to active learning. In general, participants received 45 minutes of extra physical activity daily as part of the curriculum, totaling an additional 225 minutes of PA per week. **TIME:** 4 years	1,221 (11–12 years old)	2015–2019	**INSTRUMENT:** Self-Reported Strengths and Difficulties Questionnaire (SDQ-S). **ANALYSES:** Mixed linear model, Spearman rank-order correlation, and *t*-tests.	No effect was found after a 4-year school-based PA intervention on psychosocial health problems. The adjusted effect within the intervention schools showed a borderline significant increase in the total difficulty scores for PA participation between 2018 and 2019 (mean difference: 1.02, 95% CI= –1.82 to –0.23, $p\leq0.01$). Educational level showed a weak negative correlation with the total difficulty score ($r=-0.1$). The group comparison analysis revealed that control schools had lower SDQ-S scores each year during the intervention.

**17**	Verswijveren, S. J. J. M. *et al*. (2022). Australia	Self-efficacy, lack of support, lack of self-regulation strategies, and lack of enjoyment	**INTERVENTION:** "RAW-PA": Based on social cognitive theory and behavioral choice theory, aimed at increasing physical activity through the combination of a wearable activity tracker with digital resources provided through a private Facebook group. The intervention combined the Fitbit Flex and its accompanying app with individual or weekly interactive missions, including behavior change resources (e.g., infographics, videos, and social forums), which were accessible through a private, researcher-moderated Facebook group with alerts for new content at student-scheduled times. **TIME:** 3 months	273 (≥13 years old) physically inactive	2021	**INSTRUMENT:** Questionnaires ("Physical Activity Perceived Barriers and Benefits Scale" and "Physical Activity Enjoyment Scale") and accelerometry. **ANALYSES:** Mixed linear models.	No effect from the intervention was observed on any of the remaining identified potential mediators, despite the intervention aiming to alter the target variables [self-efficacy (p = 0.76); peer support (p = 0.99), family support (p = 0.14), teacher support (p = 0.48); self-regulation (p = 0.23) and enjoyment (p = 0.67)]. The intervention group perceived more barriers to physical activity than the waitlist control group at the 6-month follow-up (adjusted mean difference = 1.77; 95% CI 0.19-3.34; p = 0.03).
**18**	Wilson, D. K. *et al*. (2011). Columbia	Lack of behavioral and social and environmental skills	**INTERVENTION:** "Active by Choice Today" combined the theories of Social Cognitive Theory and Self-Determination Theory to promote behavioral and socio-environmental skills. Physical Activity and Motor Vigor (AFMV) activities (60 min) included weekly selection of a behavioral and motivational skills component (30 min). **TIME:** 17 weeks	1,563 (10–14 years) (55% women)	2008-2009	**INSTRUMENT:** Self-reportedomni-directional, questionnaire, Intrinsic Motivation Inventory and Actical accelerometers (Mini-Mitter, Bend, OR). **ANALYSES:** Mixed ANCOVAs and Multiple imputation.	Students in the ACT intervention engaged in 4.87 more minutes of AFMV per day (95% CI=1.18–8.57) compared to those in the control condition. The effect size for this increase throughout the week was Cohen's *D*=0.24. The ICC for AFMV at the midpoint of the intervention was 0.024, indicating an increase of 27 minutes per week in AFMV. There was an increase in enjoyment (*B*=0.69, SE= 0.29, *df*=22; *p*<0.05); greater choice of activities (*B*=0.69, SE=0.29, *df*=22; *p*<0.05), with Cohen's *D* effect sizes of 0.32 and 0.29.
**MIXED**							
**19**	Carlin, A. *et al*. (2018); e **Carlin, A. *et al*., (2019). North Ireland	Lack of motivation, lack of time, and lack of opportunities in the school setting	**INTERVENTION:** Structured walking sessions, lasting 10 to 15 minutes, led by peers during the school week, part of the "Walking in Schools" program. The intervention content was developed using Social Cognitive Theory (SCT) and exposed participants to various influences on self-efficacy. Participants also received reminder cards from the research team containing tips and general advice regarding brisk walking and	199 girls [45 (11–14 years old) in complementary study]	2014–2015	**INSTRUMENT:** Interviews, accelerometer, Queens College Step Test, Evenson Actigraph cut-off points, questionnaire, and perceived benefits and barriers to exercise scale. **ANALYSES:**	The intervention increased the daily light-intensity physical activity (PA) behavior in these adolescents but did not change moderate to vigorous physical activity (MVPA). Significant differences were observed for the total average time of use (min/week) between the intervention (3715.32 min/week) and control (3081.36 min/week) groups (*p*=0.002). The main effect comparing both groups was significant, with those in the intervention group increasing total daily school-time PA by 9.2 minutes/day compared to an increase of 1.2 minutes/day in the control group

			information on how to set goals. After each walking session, participants received a reward stamp that could be accumulated and exchanged for small prizes and rewards.<br><br>**TIME:** 12 weeks			Thematic analysis, mixed ANOVA, post hoc tests, and chi-square test.	($F(1,115)$= .74, p=0.007, partial eta squared (n2)=0.061, moderate effect size). A significant group × time interaction was observed for light-intensity PA across the school day ($F(1,115)$=9.30, p=0.003, n2=0.075, moderate effect size), with a significant difference also observed between groups (increased by 8.3 minutes/day among intervention participants, compared with a decrease of 2.1 minutes/day among control participants) ($F(1,115)$=5.80, p=0.018, n2 = 0.048, small effect size). Additionally, complementary data from this study demonstrated the importance of social support in encouraging adolescent girls to be more active. Participants also highlighted that walking during school hours can overcome some barriers such as lack of time, company, and encouragement.
**20**	Corder, K. *et al*. (2020).<br><br>North Ireland	Lack of support, motivation, time pressure, and self-esteem	**INTERVENTION:**<br>"GoActive" with activities both in and out of school, focusing on increasing peer support, self-efficacy, self-esteem, and friendship quality. It was implemented in tutor groups using a hierarchical leadership system led by students, following an evidence-based interactive approach. Each tutor group (9th grade class or classroom) selected 2 activities per week from a provided selection.<br><br>**TIME:** 12 weeks	1,874 (13–14 years old)	2016–2017	**INSTRUMENT:**<br>Self-reported questionnaires, accelerometers, and interviews.<br><br>**ANALYSES:**<br>Analysis of covariance, linear regression model.	The average MVPA (moderate-to-vigorous physical activity) assessed by the accelerometer decreased in both random groups between baseline and the 10-month follow-up [–1.91 (–5.53, 1.70)]. Inconclusive subgroup analyses suggested a negative intervention effect for boys (p=0.022) and a positive effect for those with low to medium socioeconomic status (p=0.005). Overall, 62.9% of students and 87.3% of mentors reported that GoActive was fun.
**21**	Corepal, R. *et al*. (2019).<br><br>North Ireland	Lack of motivation, support, and mental malaise	**INTERVENTION:**<br>Pedometer competition "The StepSmart Challenge" to stimulate physical activity , combined with self-determination theory through goal setting, monitoring, and immediate feedback.<br><br>**TIME:** 22 weeks	224<br>(12–14 years)	2015	**INSTRUMENT:**<br>Questionnaires, ActiGraph accelerometer, and interviews.<br><br>**ANALYSES:**<br>Descriptive analyses, median and interquartile ranges (IQR), and thematic analysis.	The daily minutes of moderate-to-vigorous physical activity (MVPA) for the intervention group remained unchanged from baseline at T1 and T2. In the control group, there was a slight increase from baseline in MVPA at T1 (47.4 min/day; IQR 32.7 to 65.1), which decreased closer to baseline levels at T2 (37.2 min/day; IQR 26.5 to 53.1). The team-based competition appeared to be an acceptable approach to encourage physical activity among participants, promoting participation.
**22**	Dudley, D. A. *et al*. (2010).<br><br>Australia	Lack of enjoyment when participating	**INTERVENTION:**<br>Sports program with new activities, such as yoga/pilates/dance sessions conducted in the classroom using instructional	38 low-income girls (11th grade)	2005–2006	**INSTRUMENT:**<br>Interviews, self-reported questionnaires, and classroom observations.	The girls in the intervention group, compared to the control group, showed greater improvement in their enjoyment of physical activity during school sports (adjusted mean difference = 3.8, 95% CI = –2.4 to

		in PA, lack of support, and low self-perception	videos, an introductory tennis training course, and water games in a pool, based on Social Cognitive Theory. The focus was on the interaction between personal, behavioral, and dynamic factors.<br><br>**TIME:** 3 months			**ANALYSES:**<br>Analysis of covariance, independent samples *t*-tests, and Cohen's.	10.1; Cohen's *d*=0.42 standard deviation units) and body image (adjusted mean difference = 1.0, 95% CI = –0.4 to 2.3; *d*=0.50). Additionally, there was a smaller decline in participation in physical activity during school sports (adjusted mean difference = 13.6, 95% CI= –21.8 to 48.9; *d*=0.24). Overall, the intervention participants reported that the school sports program helped increase their enjoyment of physical activity.
**23**	Ferreira Silva, R. M. *et al*., (2023).<br><br>Brazil.	Lack of knowledge, lack of encouragement, and lifestyle	**INTERVENTION:** Sending eight illustrated and colored folders was based on the strategy developed by the group 'On Your Feet Britain (10 Ways to Sit Less at Work)' and an intervention carried out with university students. It also drew on activities described in the Physical Activity Guide for the Brazilian Population, designed for children and young people aged 6 to 17, across the domains of leisure time, commuting, school, and household chores.<br><br>**TIME:** 4 weeks	80 (15.9±1.15 years)	2021	**INSTRUMENT:**<br>Two questionnaires and a structured interview<br><br>**ANALYSES:**<br>Descriptive and inferential statistics, *t*-test, and content analysis.	The intervention was not effective in increasing physical activity levels or significantly reducing time spent in sedentary behavior among adolescents. Regarding the time exposed to sedentary behavior, a reduction in the meantime of 47.14 min per day in the IG and an increase in the meantime of 31.37 min per day in the CG was observed.
**24**	Koorts, H. *et al.* (2020).<br><br>Australia	Lack of awareness, motivation, and incentive	**INTERVENTION:**<br>"Raising Awareness of Physical Activity"; an electronic device and app that tracks activities, provides weekly interactive goals or missions, motivational videos, and forums.<br><br>**TIME:** 12 weeks	142 (mean age 13.7 years)	2016–2018	**INSTRUMENT:**<br>Questionnaire and interviews.<br><br>**ANALYSES:**<br>Descriptive statistics, Mann-Whitney test, and thematic analysis.	Adolescents reported that the Fitbit Flex increased their motivation (85/120, 70.8%) and awareness (93/119, 78.2%) regarding physical activity. Despite low adherence and engagement, increased motivation to be active due to peer competition was often mentioned as having a positive impact on their physical activity (p = 0.05).
**25**	Kroshus, E. *et al*., (2023).<br><br>USA	Lack of suitable environments and lack of company	**INTERVENTION:**<br>Distribution of "play kits" (activity kits) including balls (basketball, soccer, volleyball), jump ropes, resistance bands, and activity sheets with ideas to promote physical activity (PA) before and after school, both individually and with family.<br><br>**TIME:** 12 months	1,076<br>(11–14 years old)	2020	**INSTRUMENT:** Self-reported questionnaire and interviews<br><br>**ANALYSES:**<br>Descriptive analysis, chi-square tests, and thematic analysis.	At the intervention school, students were active for at least 60 minutes an average of 3.14 days per week (95% CI=2.75–3.53) when not enrolled in PE, and 3.60 days (95% CI=3.21–4.00) when enrolled in PE. Most students reported that the play kit motivated them to participate in PA, gave them activity ideas, and made virtual PE more enjoyable. In both the intervention and control schools, there was no difference over time in PA efficacy, enjoyment, PE enjoyment, or level of PA.

**26**	Lubans, D. R. *et al*. (2014).<br><br>Australia	Lack of motivation and long screen time	**INTERVENTION:**<br>"Active Teen Leaders Avoiding Screen Time," involving teacher professional development, investigator-led seminars, enhanced school sports sessions, lunchtime physical activity guidance sessions, provision of fitness equipment to schools, pedometers for self-monitoring, parental strategies to reduce screen time, and a smartphone app and website.<br><br>**TIME:** 20 weeks	361 (12.7±0.5 years old) inactive boys	2012–2013	**INSTRUMENT:**<br>App evaluation interviews and questionnaires<br><br>**ANALYSES:**<br>Descriptive and inductive analysis	Focus group participants reported enjoying the program and felt it provided them with new skills, techniques, and routines for the future. Additionally, the push notifications reminded them to be more active and reduce screen time. Participants' intentions to limit recreational screen time (mean = 3.95±1.07), engage in regular MVPA (mean = 4.16±0.81), and participate in muscle-strengthening activities (mean = 4.08±0.76) were high after completing the program.
**27**	Moore, R. *et al*., (2024).<br><br>Inglaterra	Lack of confidence and motivation	**INTERVENTION:**<br>Through a conversational agent (CA) named "Phyllis," using theory-based approaches, a channel (school website or social media) provided support to overcome barriers to physical activity (PA) and offered information and guidance on how to be physically active for one hour. The prototype included two modules focused on motivation and confidence. The CA identified barriers to PA based on user input. In addition to addressing these barriers, it provided solutions, including recommendations for activities they could participate in.<br><br>**TIME:** 3 months	9 (11–13 years old)	2023	**INSTRUMENT:**<br>"AttrakDiff" questionnaire, workbook, and interviews<br><br>**ANALYSES:**<br>Basic frequencies and thematic analysis	Concerning acceptability, a sizable portion of the students (34/46, 73%) expressed their definite intention to use a fully functional CA in the future. A total of 80% (37/46) of students reported being “confident in their ability to engage in PA or play sports,” indicating a 50% increase from the initial pre-response rate of 53%. The modules designed for the CA showed promising results, promoting greater confidence and motivation for PA. Promising outcomes were observed, with increasing confidence and motivation for PA.
**28**	Sutherland, R. *et al*. (2020).<br><br>Australia	Lack of support	**INTERVENTION:**<br>The PA4E1 program includes seven physical activity (PA) practices: physical education classes, activity plans, school sports programs, PA during recess, school PA policies, community PA providers, and communication with parents.<br><br>**TIME:** 24 months	6,476; 49 schools from grades 7th to 9th	2017–2019	**INSTRUMENT:**<br>Blocks and interviews<br><br>**ANALYSES:**<br>Descriptive statistics, chi-square test, and logistic regression model	The multicomponent implementation support intervention led to an increase in the number of PA practices implemented by secondary schools. After 12 months, more schools in the program group implemented four out of the seven PA practices (17/24, 70.8%) compared to the control group (1/25, 4%) (p<0.001), with an average of 3.2 (2.4–3.9) more practices (p<0.001, mean 3.9 (SD=1.5) vs. 0.7 (SD=1.0)).

			**QUALITATIVE**				
**29**	Bean, C. N. *et al.* (2014).<br><br>Canada	Lack of support, lack of suitable environments and structures, lack of self-control and motivation	**INTERVENTION:**<br>The "Girls Just Wanna Have Fun" program was implemented through workshops, awareness lectures, and a series of sessions aimed at promoting (1) Personal Responsibility or Self-Control; (2) Effort; (3) Self-Coaching; (4) Leadership; and (5) Transferability.<br><br>**TIME:** 12 months	10 girls (11–14 years old)	2011–2012	**INSTRUMENT:**<br>Interviews and logbook<br><br>**ANALYSES:**<br>Inductive thematic analysis	The program had a positive impact on participants while also fostering greater autonomy. For instance, the "Rose and Thorn" activity proved to be an effective way of connecting with others and positively influenced the lack of motivation and support. The program succeeded in facilitating PA and provided opportunities for young people who might not have otherwise experienced such practices. As one participant reported: *"It made me more active and, for some reason, it made me happy, and I thought, 'Oh, yes, I'll have something to do after school!' And my mom is happy because I'm more active" (Y-3).*
**30**	Drehlich, M. *et al.* (2020).<br><br>Australia	Lack of motivation and socio-economic conditions	**INTERVENTION:**<br>Through missions on a wearable activity tracker (Fitbit Flex) supported by digital materials delivered via social media (Facebook).<br><br>**TIME:** 12 weeks	124 physically inactive (13–14 years old)	2020	**INSTRUMENT:**<br>Interviews<br><br>**ANALYSES:**<br>Content analysis	The Fitbit Flex was helpful for tracking physical activity and motivating participants to engage in physical activities. However, perceptions regarding its ease of use were often negative, leading to low acceptance and engagement with the technology.
**31**	Lodewyk, K. R. *et al.* (2023).<br><br>Canada	Lack of motivation	**INTERVENTION:**<br>The "Intramural Program Planning" intervention applied 25 student facilitators (Intrapersonal, Interpersonal, and Environmental) to enhance motivation and participation in physical activities. This was achieved through intramural leadership training, multiple peer group planning sessions, and subsequently organizing and participating in engaging activities.<br><br>**TIME:** 3 months	25 students girls (of 483)	2023	**INSTRUMENT:**<br>Interviews<br><br>**ANALYSES:**<br>Content analysis	There was a noticeable reduction in the perceived barriers for women to engage in physical activities at school following the program intervention. The affirming atmosphere provided a positive alternative and often helped motivate friends to participate.
**32**	Mitchell, F. *et al.* (2015).<br><br>Scotland	Lack of motivation, autonomy, and interest	**INTERVENTION:**<br>"Fit for Girls" was based on self-determination theory, focusing on three areas: competence, relatedness, and	41 girls (11–16 years old)	2008–2011	**INSTRUMENT:**<br>Interviews<br><br>**ANALYSES:**	The PA intervention, which included consultation and activity choice, resulted in increased participation and more positive perceptions of the subject for many girls. These factors created a more favorable physical

			autonomy, over three years. PE teachers participated in training workshops. After the workshop, each school developed an action plan to address specific areas of intervention. Consequently, the intervention content and its implementation varied according to the school's specific needs.<br><br>**TIME:** 36 months			Thematic and content analysis	education environment and were crucial in transitioning the girls from disengagement to engagement. The primary reason for such changes in the girls' attitudes and behaviors was the new PE environment fostering feelings of autonomy (by offering choices), competence (girls could choose activities in which they felt capable), and relatedness (consultation improved relationships with teachers, and the girls could better relate to peers in same-sex classes).
**33**	Pierre, S.T. *et al.*, (2024).<br><br>USA	Lack of support and encouragement	**INTERVENTION:**<br>The Up2Us Sports SBYD program, implemented in schools through physical education classes, elective courses, and other school activities, included not only coaching skills but also contextualized sessions on nutrition and physical activity. Up2Us Sports provides extensive training for its coaches on creating an inclusive environment and building strong mentoring relationships with youth from all backgrounds and fitness levels.<br><br>**TIME:** 3 years	14 (14.8±1.7 years)	2020–2022	**INSTRUMENT:**<br>Interviews<br><br>**ANALYSES:**<br>Content analysis using NVivo 12 (QSR International)	There was an increased opportunity for physical activity facilitated by peer relationships. The presence of supportive coaches helped encourage physical activity among youth, even in the context of external challenges. The program also helped foster skills such as self-confidence, impulse control, and social competence among the participants.
**34**	Wright, P. M. & Burton, S. (2008).<br><br>USA	Lack of autonomy, stress, lack of motivation, and skills	**INTERVENTION:**<br>A responsibility-based physical activity program was integrated into an intact high school physical education class aimed at: (a) establishing a relevant curriculum, (b) overcoming barriers, (c) practicing life skills, (d) recognizing transfer potential, and (e) creating a meaningful program. In the initial lessons, instruction was teacher-led, focusing on self-control and participation. After several lessons, the lead instructor integrated opportunities for group decision-making related to lesson content and organization. By the sixth lesson, students were given the opportunity to lead warm-up exercises or tai chi movements. Around the program's midpoint, discussions on goal setting, life	23 African Americans (mean age of 14.8 years) from an urban high school	2008	**INSTRUMENT:**<br>Interviews and observations<br><br>**ANALYSES:**<br>Deductive and inductive analysis	The tai chi content and the TPSR framework effectively integrated with the Lifelong Wellness curriculum by addressing physical, emotional, and mental health issues.<br>Since tai chi practice incorporates physical and mental relaxation as well as martial arts applications, it facilitated discussions on stress reduction, coping, and conflict resolution. Authentic assessment strategies, such as self-administered and peer-administered skill tests, along with the daily responsibilities journal, fostered confidence and accountability among students.<br><br>Students reported applying the lessons on stress reduction in their daily lives. The program promoted a positive learning environment and positively influenced student behavior.

			skills, and stress reduction were incorporated. Students were allotted class time to discuss and write about these topics.<br><br>**TIME:** 12 months				

Note: PA stands for physical activity, and HDI stands for human development index. ** Article published in a different year but complementary to another article by the same lead author, both referring to the same study.
