# Supplementary material for: Interventions on Barriers to the Participation of Adolescents in Physical Activity: A Systematic Review
Source: Int J Environ Res Public Health. 2025 May 31;22(6):881. doi: 10.3390/ijerph22060881 (PMC12193246; doi:10.3390/ijerph22060881)
Supplement: Supplementary file 1 [file ijerph-22-00881-s001.zip › S3 File.pdf]

PRISMA 2020 Checklist

Section and Topic	Item #	Checklist item	Location where item is reported
**TITLE**			
Title	1	Interventions on barriers to the participation of adolescents in physical activity: A systematic review	1
**ABSTRACT**			
Abstract	2	This review aimed to assess the effectiveness of interventions used to minimize barriers to participation in physical activity (PA) among adolescents. This systematic literature re-view followed the Preferred Reporting Items for Systematic Reviews and Meta-Analyses 2020 guidelines. Searches were conducted across five databases: PubMed, SPORTDiscus, Embase, Scopus, and Web of Science. Thirty-four studies evaluated interventions to over-come barriers to participation in PA, with a focus on lack of motivation, encouragement, and support, as well as intrapersonal, interpersonal, and environmental barriers. Most interventions were conducted in developed countries, with an emphasis on girls, reflecting efforts to address health inequities. The interventions, conducted in schools, included workshops, training programs, lectures, goal setting, and practical activities, lasting from four weeks to four years. Twenty-seven studies (79.4%) reported positive impacts on PA participation, particularly in interventions addressing psychosocial, psychological, and behavioral barriers. The most effective interventions combined theory (through education-al approaches) and practice (practical PA activities). These findings contribute to under-standing barriers leading to physical inactivity and provide insights for developing or replicating initiatives to improve PA levels among adolescents.<br>Keywords: primary prevention; obstacles; participation in physical activity; physical inactivity; adolescence.<br>Ethics and disclosure: Ethical approval was not required for this study as it is an analysis of previously published articles (i.e., secondary data). The results will be published in a peer-reviewed journal.<br>PROSPERO registration No. CRD42022382174.	1
**INTRODUCTION**			
Rationale	3	This review is particularly relevant considering that physical inactivity is a public health concern requiring prevention, particularly among adolescents. Beyond the significant potential as a strategy to effectively promote PA, this review aims to provide guidance to reduce health disparities and enhance overall public health [42]. Finally, the findings aim to advance the scientific knowledge of effective interventions while supporting institutional managers and the academic community in developing or replicating projects, programs, and initiatives to promote PA among adolescents.	3
Objectives	4	This review aimed to assess the effectiveness of interventions designed to minimize barriers to participation in physical activity (PA) among adolescents. It addresses two research questions: “What are the characteristics and outcomes of interventions implemented to minimize barriers to PA?” and “What are the most commonly recurring barriers encountered during the implementation of initiatives to reduce barriers to PA?”.	3
**METHODS**			
Eligibility criteria	5	The search strategy and eligibility criteria followed the PICO (Population, Intervention, Comparison, Outcome) [45–47], which is widely used in evidence-based healthcare research. The “P” component included terms representing adolescents aged 10-19 years of both sexes, as defined by the WHO [9]. The “I” component included terms related to interventions aimed at reducing barriers to participation in PA. The “C” component referred to the absence of interventions and the “O” component focused on participation in PA.<br>For this review, original peer-reviewed studies published in English, with no date re-strictions, were eligible for inclusion, provided they implemented interventions aimed at reducing barriers to physical activity in adolescents (aged 10 to 19 years) [9]. The validity of the eligible studies was assessed, and any retraction records were identified using the Scite tool [48].<br>Interventions conducted in clinical settings (hospitals and/or nursing homes) and those focusing solely on specific populations (such as rural, Indigenous, refugee, and isolated groups) were excluded. Studies with incomplete data, opinion articles, case reports, commentaries, editorials, dissertations, theses, reviews, and cross-sectional studies were also excluded, as well as those that were inaccessible even after attempts to contact the authors. Similarly, studies involving adolescents with physical or mental disabilities or chronic diseases during sampling were excluded, as well as those that included age groups outside adolescence, except when data were presented separately or could be calculated. Additionally, duplicates published in more than one journal were carefully re-viewed to avoid redundancies, and studies	4

Section and Topic	Item #	Checklist item	Location where item is reported
		that included retractions were also excluded. Finally, studies that discussed barriers to physical activity without the implementation of an associated intervention were excluded, and these could only be identified after a full reading of the articles. All exclusion criteria were carefully outlined in the protocol [43].<br>After extracting metadata from the databases through the search strategy, the results were imported into EndNote™ X9 software (Clarivate, Philadelphia, PA, USA) to identify and remove duplicates [49]. The review process followed three steps: 1) screening of titles and abstracts; 2) study selection based on eligibility criteria; and 3) full-text review of potentially eligible studies. Two independent researchers (LFT and LMTR), who were trained to screen articles, performed all steps using Rayyan software (Rayyan Systems Inc., Cam-bridge, MA, USA) [50]. To ensure accuracy, the reviewers conducted a cross-check of eligibility, and any discrepancies in study selection were resolved by a third reviewer (PRES), enhancing the reliability of the selection process. At the end of this process, the articles were included in the systematic review. Inter-rater reliability was calculated at each phase using GraphPad® (GraphPad Software, LLC, San Diego, CA, USA), available at: https://www.graphpad.com/quickcalcs/kappa2/.	
Information sources	6	Searches were conducted in five databases: MEDLINE/PubMed® via the National Library of Medicine® interface, SPORTDiscus® via the EBSCOhost™ interface, Embase™, Scopus™, and Web of Science Core Collection™ between February 1, 2023, and August 1, 2024.	4
Search strategy	7	1 = "physical activity" OR "sedentary lifestyle" OR "physical inactivity" OR "sedentary behavior"<br>2 = "adolescence" OR "adolescent" OR "college students" OR "high school student" OR "student" OR "teen" OR "teenagers" OR "undergraduate student" OR "university student" OR "youth"<br>3 = "barriers" OR "challenges" OR "difficulties" OR "obstacles"<br>4 = 1 AND 2 AND 3, in English, with no restrictions on publication date.	Suppplementary (Table S1 and S1 File)
Selection process	8	Two independent researchers (LFT and LMTR), who were trained to screen articles, per-formed all steps using Rayyan software (Rayyan Systems Inc., Cambridge, MA, USA) [50].	4
Data collection process	9	The review process followed three steps: 1) screening of titles and abstracts; 2) study selection based on eligibility criteria; and 3) full-text review of potentially eligible studies.	4
Data items	10a<br>10b	The following data were extracted and summarized from the articles included in the systematic review (S2 File): i) author, year, and place or country of study, ii) barriers, iii) intervention used and time, iv) sample, v) period in which data collection occurred, vi) type of study, vii) instrument used for data collection, viii) type of analysis performed, and ix) main results.	5 and Supplementary S2 File
Study risk of bias assessment	11	The strength of evidence was assessed using the Grading of Recommendations, Assessment, Development, and Evaluations (GRADE) questionnaire [53,54] in GRADEpro GDT online software (McMaster University and Evidence Prime, Inc., Hamilton, ON, Canada). The GRADE criteria evaluate risk of bias, inconsistency of results, indirectness of evidence, imprecision, and publication bias in healthcare research [55]. Quality of evidence was categorized as: (a) high, (b) moderate, (c) low, or (d) very low [55].<br>To assess the risk of bias in quantitative studies, the translated and adapted version of the 27-item Downs and Black [56]. However, as some items of the questionnaire were not applicable to observational studies, a modified, condensed version (0-16 points) for longitudinal studies was used [57]. Thus, a group of 16 questions (corresponding to Questions 1–3, 5–7, 9–12, 17, 18, 20, 21, 25, 26) was selected for use. Quality scores were calculated for each study and expressed as a percentage of the maximum possible score for the study design [56]. Studies scoring 70% or higher were classified as having a 'low risk of bias', while scores below 70% were considered to indicate a 'high risk of bias' [53,54].<br>The Critical Appraisal Skills Programme Qualitative Research Checklist (CASP) [58] was used to assess the risk of bias in qualitative studies. The evidence was evaluated through ten criteria [59]: 1) clear objectives; 2) methodology appropriate to the objectives; 3) study design appropriate to the objectives; 4) appropriate recruitment strategy; 5) data col-lection methods appropriate to the research question; 6) researcher-participant relation-ship; 7) ethical considerations; 8) rigorous analysis; 9) clear presentation and discussion of results; and 10) research contributions and implications to scientific knowledge. Studies were categorized based on their scores as: low (0-3 points), moderate (4-7 points), or high quality (8-10 points) [60].	5 and 6

Section and Topic	Item #	Checklist item	Location where item is reported
Effect measures	12	Not applied.	-
Synthesis methods	13a	Two independent reviewers (LFT and LMTR) assessed the strength of evidence and risk of bias, with discrepancies resolved by a third reviewer (PRES). The reviewers under-went preparatory training [61], and all methodological procedures adhered to the pre-registered protocol [43]. All studies were examined for declarations of potential conflicts of interest and ethical approval.	6
	13b	Two independent reviewers (LFT and LMTR) synthesized the results, with discrepancies resolved by a senior third reviewer (PRES).	6
	13c	Table 1, 2, 3 and 4, Figure 2, Figure 3, S2 File, Figure S1.	7-12
	13d	To aggregate intervention outcomes (S2 File) and present them (Table 2), studies were analyzed based on their proposals, considering the main results and conclusions presented in each study. All studies were categorized into three levels of intervention effectiveness, was based on Table 3: 'Low' for studies showing no significant increase or negative out-comes in PA - considered an ineffective result, as indicated in Table 3; 'Moderate' for those demonstrating limited or inconsistent positive effects (these studies may have addressed relevant barriers, but the intervention effectiveness was not sufficiently strong) - considered an inconsistent result (showing effects but lacking strong evidence) as indicated in Table 3; and 'High' for interventions achieving statistically significant improvements in PA ($p < 0.05$ in most studies) and consistent improvements in the levels of PA of the participants - considered a successful result (Table 3), main intervention outcomes are presented in the extraction table (S2 File). A meta-analysis was not conducted due to substantial heterogeneity in study designs, outcome measures, and intervention types, which precluded statistical pooling of results.	5
	13e	Not applied.	-
	13f	Not applied.	-
Reporting bias assessment	14	After the full-text screening is completed, the articles included will be assessed for quality using the recommendations (GRADE) and Critical Appraisal Skills Program Qualitative Research Checklist (CASP), Downs and Black (D&B). The risk of bias will be assessed independently by two reviewers.	6
Certainty assessment	15	Not applied.	-
**RESULTS**			
Study selection	16a	The study identification, selection, and evaluation followed the Preferred Reporting Items for Systematic Reviews and Meta-Analyses (PRISMA) 2020 guidelines [31].	3
	16b	The search strategy and eligibility criteria followed the PICO (Population, Intervention, Comparison, Outcome) [32–34], which is widely used in evidence-based healthcare research. The “P” component included terms representing adolescents aged 10-19 years of both sexes, as defined by the WHO [8]. The “I” component included terms related to interventions, actions, and programs aimed at reducing barriers to participation in PA. The “C” component referred to the absence of interventions and the “O” component focused on participation in PA.<br>Literature reviews, cross-sectional studies, and studies with samples including adolescents with physical or mental disabilities or chronic diseases were excluded. Additionally, interventions in hospital settings, those addressing only specific cohorts (rural, indigenous, refugees, or isolated), and studies with incomplete data were excluded. All exclusion criteria were carefully outlined in the protocol [30].	4
Study characteristics	17	Table 1, 2, 3, 4, S2 File, Figure 2, 3 and S1 Table.	8-16
Risk of bias in studies	18	Table 4	12
Results of individual studies	19	Table 1, 2, 3, 4, S2 File, Figure 2, 3 and S1 Table.	8-16
Results of	20a	Table 1, 2, 3, 4, Figure 2, 3 and S2 File.	8-16

Section and Topic	Item #	Checklist item	Location where item is reported
syntheses	20b	Not applied.	-
	20c	Not applied.	-
	20d	Not applied.	-
Reporting biases	21	Not applied.	-
Certainty of evidence	22	Not applied.	-
**DISCUSSION**			
Discussion	23a<br>23b<br>23c<br>23d	This systematic review synthesizes the findings of interventions aimed at reducing barriers to PA participation among adolescents. The included studies, conducted in 17 countries and involving more than 17,000 adolescents, evaluated programs addressing intrapersonal, interpersonal, and environmental barriers, including lack of time, insufficient knowledge, lack of parental support, absence of suitable environments, and inadequate facilities and equipment. The most effective interventions combined educational elements (e.g., theoretical instruction) with practical PA. Finally, most interventions were conducted in developed countries, with an emphasis on girls, reflecting efforts to reduce health inequalities.<br>This review highlighted several studies that contribute to the development and replication of effective interventions to increase PA. However, there are still opportunities to improve the design and reporting of these studies. The novelty of this review lies in com-piling scientific evidence on interventions aimed at reducing barriers to participation in PA among adolescents. This compilation identified gaps in literature and informed future studies. A notable strength is the use of rigorous, validated, and well-established methods, along with reviewer training. This systematic process enabled the exclusion of retracted studies.<br>Additionally, the limitations of the included studies, often overlooked in evaluations, were reported. However, this study also has limitations. Firstly, despite reviewer training, disagreements arose due to the subjective nature of interventions addressing PA barriers, particularly in the initial review phase. This was largely due to exclusion criteria that did not account for studies, identified as ineligible, only after a full-text review [136]. Second, the exclusion of grey literature in the review may have resulted in relevant information being missed. Methodological heterogeneity among studies prevented a meta-analysis, as they differed in design, with similar barriers, both in terms of timing and methodology, and even in execution. Additionally, there was a lack of standardization in assessing intervention effectiveness. Future studies should apply rigorous methodologies, including bias control and context-sensitive approaches, ensuring durations longer than three months but under a year. Identifying barriers through targeted surveys and refining existing interventions, even those with inconsistent results, will help generate more reliable evidence.	12-16
**OTHER INFORMATION**			
Registration and protocol	24a<br>24b<br>24c	This systematic review protocol was registered in PROSPERO (No. CRD42022382174) and subsequently published [30]. The study identification, selection, and evaluation followed the *Preferred Reporting Items for Systematic Reviews and Meta-Analyses* (PRISMA) 2020 guidelines [31]. Ethical approval was not required, as only published articles were analyzed.	3
Support	25	This work was supported by the Federal Institute of Goiano through an undergraduate research grant.	16
Competing interests	26	The authors have declared that no competing exists.	16
Availability of data, code and other materials	27	File S1: Search strategy; File S2: Main version of the table used to extract data from the included studies; File S3: Checklist of the PRISMA 2020; Table S1: Keywords comprising the search strategy organized in blocks.	16
